# Co-regulated gene expression of splicing factors as drivers of cancer progression

**DOI:** 10.1038/s41598-019-40759-4

**Published:** 2019-04-02

**Authors:** Esmee Koedoot, Marcel Smid, John A. Foekens, John W. M. Martens, Sylvia E. Le Dévédec, Bob van de Water

**Affiliations:** 1 Division of Drug Discovery and Safety, LACDR, Leiden University, Leiden, The Netherlands; 2 Department of Medical Oncology and Cancer Genomics Netherlands, Erasmus MC Cancer Institute, Erasmus University Medical Center, Rotterdam, The Netherlands

## Abstract

Splicing factors (SFs) act in dynamic macromolecular complexes to modulate RNA processing. To understand the complex role of SFs in cancer progression, we performed a systemic analysis of the co-regulation of SFs using primary tumor RNA sequencing data. Co-regulated SFs were associated with aggressive breast cancer phenotypes and enhanced metastasis formation, resulting in the classification of Enhancer- (21 genes) and Suppressor-SFs (64 genes). High Enhancer-SF levels were related to distinct splicing patterns and expression of known oncogenic pathways such as respiratory electron transport, DNA damage and cell cycle regulation. Importantly, largely identical SF co-regulation was observed in almost all major cancer types, including lung, pancreas and prostate cancer. In conclusion, we identified cancer-associated co-regulated expression of SFs that are associated with aggressive phenotypes. This study increases the global understanding of the role of the spliceosome in cancer progression and also contributes to the development of strategies to cure cancer patients.

## Introduction

To maintain the complexity of intracellular as well as extracellular homeostasis, cells require a vast number of well-controlled biological programs. To keep these programs in balance, a tight regulation of the transcription and translation of the various components of these programs is essential. One of the critical processes involved in this regulation is splicing: the exclusion of non-coding pre-messenger RNA (pre-mRNA) regions (or introns) resulting in a transcript that can be translated into a functional protein^1^. Moreover, by including or excluding specific exons, splicing can regulate the expression of different isoforms of the same gene, thereby providing a new layer of genetic control and diversity in biological gene function^2^. Splicing is characterized by a complex series of reactions involving different small nuclear ribonucleoproteins (snRNPs): RNA-protein complexes that can bind to pre-mRNA and various other proteins. These snRNPs comprise 5 small nuclear RNAs (snRNAs) and can associate with approximately 250 proteins that are also named splicing factors^3^.

Dysregulation of splicing is involved in a wide variety of diseases, such as muscular dystrophy, Parkinson’s disease and cardiac disease^4^. Furthermore, the flexibility to remodel the conformation and function of almost every cellular protein can be used by cancer cells in both tumor development and metastatic progression^5,6^. From this perspective, alternative splicing as well as single spliceosome components have been linked to apoptosis, regulation of oncogenes, invasion and metastasis, metabolism and angiogenesis in several cancer types, including breast cancer. For example, the splicing factor class that consists of heterogeneous nuclear ribonucleoproteins (hnRNPs) is known to control metastasis formation by regulating alternative splicing of the small GTPase Rac1^7^, but also by affecting CD44 isoform expression which increases TGFβ signaling^8^. Other hnRNP group members hnRNPA1 and hnRNPA2 are involved in deregulating cellular energetics, necessary to feed the cancer cells during cell growth and division^9,10^. Finally, some splicing factors are highly mutated in cancer with SF3B1 being a driver gene in breast cancer^11^. Since the splicing machinery seems to play such a critical role in cancer development and progression, targeting specific splicing factors might provide a therapeutic window to combat cancer progression and improve patients survival rates^12^. So far, most of these studies focused on the role of single splicing factors. Yet, it should be kept in mind that splicing factors are assembled in macromolecular complexes that are dynamic in composition, time and space. Therefore, we hypothesized that subsets of splicing factors are likely co-regulated in expression and, thereby, together act in driving the modulation of specific splicing events that would promote cancer progression.

To assess our hypothesis, we made advantage of large datasets of breast tumor-derived patient RNA sequencing-based gene expression (The Cancer Genome Atlas and BASIS^11,13^). Using the two independent RNA sequencing datasets, we correlated the expression of all combinations of splicing factors and identified two subclasses of splicing factors with distinct expression behavior, which we referred to as ‘Enhancer-SFs’ and ‘Suppressor-SFs’. In breast cancer, high Enhancer-SF expression levels associate with a more aggressive tumor phenotype and higher risk of developing metastases. Remarkably, the Enhancer- and Suppressor-SF patterns are also observed in more than 30 other cancer types, including highly prevalent and aggressive cancer types such as pancreas and lung cancer. This study elicits an important role for splicing in cancer progression and might initiate the discovery of new biomarkers and treatments to combat this deadly disease.

## Results

### Co-regulated expression of splicing factors in human breast cancer

**Figure 1:**
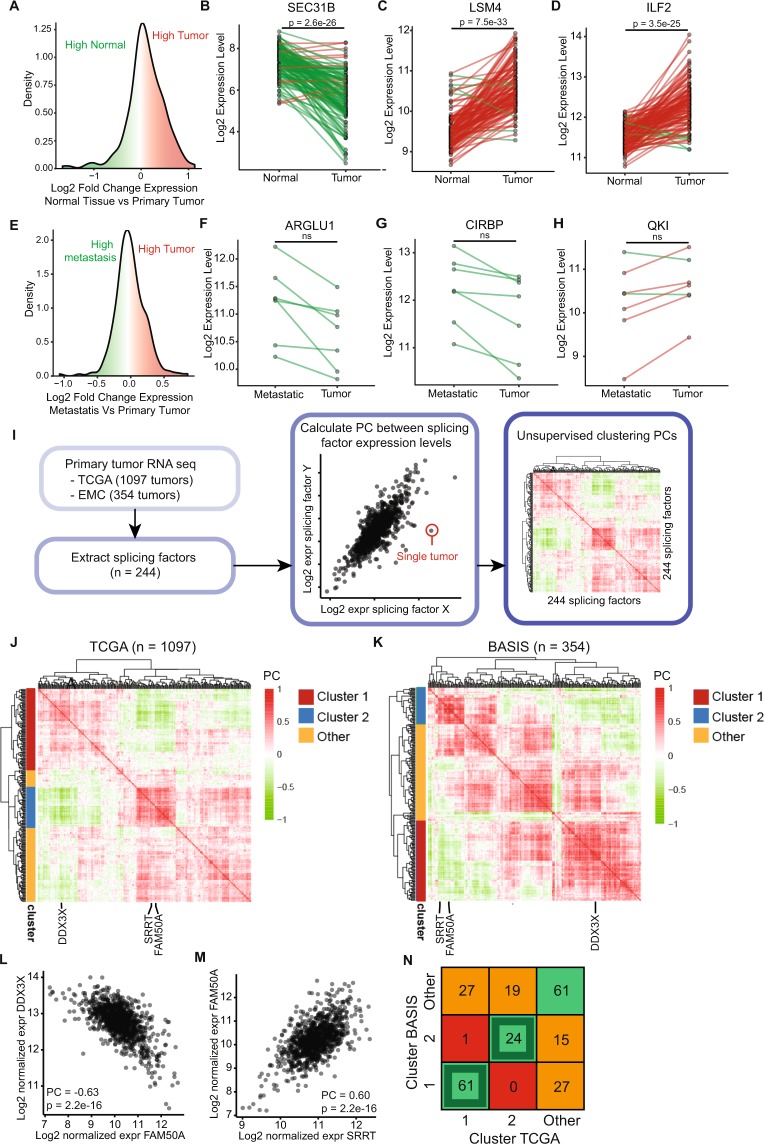
Co-regulation of splicing factors in human breast cancer. (**A**) The mean log2 fold changes between primary breast tumor and matched normal tissue was calculated for all splicing factors (n = 244) and visualized in a density plot. Log2 fold change > 0: factors were higher expressed in tumor tissue; log2 fold change < 0: factors were higher expressed in normal tissue. (**B**–**D**) Examples of splicing factors that are down (**B**) or upregulated (**C**,**D**) in tumor tissue compared to normal breast tissue. Dots represent single patients, matched patients are connected with a line (green = higher expressed in normal tissue, red = higher expressed in tumor tissue). (**E**) The mean log2 fold changes between primary breast tumor and matched metastatic tissue was calculated for all splicing factors (n = 244) and visualized in a density plot. Log2 fold change > 0: factors were higher expressed in metastatic tissue; log2 fold change < 0: factors were higher expressed in primary tumor tissue. (**F**–**H**) Example splicing factors that are down (**F**,**G**) or upregulated (**H**) in tumor tissue compared to metastatic tissue. Dots represent single patients, matched patients are connected with a line (green = higher expression in metastatic tissue, red = higher expression in primary tumor tissue). (**I**) Method used to calculate Pearson Correlation coefficients (PCs) between RNA expression levels of splicing factors. (**J**) Hierarchical clustering (Euclidean distance, complete linkage) of the correlation of splicing factor expression levels in TCGA RNA sequencing data (red = high positive correlation, green = high negative correlation). The optimal cluster number was determined using CIvalid^43^. (**K**) Same as J for BASIS RNA sequencing data (only expression data for 235 factors available). (**L**) Example of highly negatively correlating splicing factors in TCGA RNA sequencing data. Dots represent single patient tumors. (**M**) Same as K for highly positively correlating splicing factors. **N**. Number of genes that overlap between BASIS and TCGA hierarchical clusters shown in (**J**,**K**) *p < 0.05, **p < 0.01, ***p < 0.001. P-values were calculated with a paired t-test and corrected for multiple hypothesis testing using the Benjamini Hochberg method.

In order to systematically evaluate the role of the complete spliceosome during breast cancer development and progression, we compared RNA expression levels of every single splicing factor (244 in total, derived from Hegele *et al*.^3^) between normal mammary gland tissue and matched primary breast tumor (Fig. 1A–D) and between primary breast tumor and metastatic tissue (Fig. 1E–H) using matched patient RNA sequencing data from 114 and 7 patients respectively, obtained from The Cancer Genome Atlas (TCGA). This analysis revealed that the spliceosome complex as a whole is not up- or downregulated during both breast cancer development (Fig. 1A) and progression (Fig. 1E). However, as expected, RNA expression levels of individual factors can be both positive and negatively related to tumor development (Suppl. Fig. 1A, Table 1) and metastasis formation (Suppl. Fig. 1B, Table 2). The splicing factor SEC. 31B was significantly lower expressed while LSM4 and ILF2 were significantly higher expressed in tumor versus matched normal mammary gland tissue (Fig. 1B–D). Similarly, ARGLU1 and CIRBP were lower expressed in tumor compared to metastatic tissue, while QKI was higher expressed (Fig. 1F–H). Given the strong association for various splicing factors with high expression in tumor compared to normal tissue, we anticipated co-expression of splicing factors in association with cancer progression. To uncover the co-regulated gene expression of spliceosomal components, we calculated the Pearson correlation coefficient (PC) of RNA expression levels for all possible combinations of the 244 splicing factors using two independent RNA sequencing datasets acquired from primary breast tumor patient derived material: 1) the TCGA dataset comprising 1097 primary breast tumors and the BASIS dataset comprising 354 primary breast tumors^11,13^. Within both datasets all breast cancer subtypes and stages are represented, with one major difference in the proportion of HER2 positive samples (22.5% in TCGA dataset vs. 1.5% in the BASIS dataset, see Suppl. Table 3). By calculating the PCs between the transcript levels of all splicing factors (Fig. 1I), we identified pairs of factors positively (Fig. 1L) or negatively (Fig. 1M) correlating with each other. Unsupervised clustering of the PCs calculated using both RNA sequencing data from TCGA (Fig. 1J, Suppl. Fig. 2A) and BASIS (Fig. 1K, Suppl. Fig. 2B) revealed 2 separate main clusters (cluster 1 and cluster 2) containing splicing factors that positively correlated within clusters (positive PC), while negatively correlated between clusters (negative PC). The strong correlation between these selected clusters was further validated by separate clusterings (Suppl. Fig. 3). Importantly, genes within the clusters derived from the analysis of the TCGA database showed strong overlap with clusters derived from the analysis of the BASIS database (Fig. 1N), providing high confidence in the actual co-regulation of these subsets of splicing factors in breast cancer. Moreover, these correlation clusters were largely validated in a third dataset consisting of microarray data of 867 primary tumors of untreated breast cancer patients (MA-867 dataset) (Suppl. Fig. 4). Even though the entire spliceosomal complex is not up- or downregulated in breast cancer development or progression (Fig. 1A,E), we discovered subgroups that seemed to be co-regulated with each other. Cluster 1 contained 61 overlapping genes, while cluster 2 contained 24 overlapping genes (Fig. 1N, Suppl. Fig. 2). Although the splicing factors that overlapped in cluster 2 contained 25% more core splicing factors^3^ compared to cluster 1 (Suppl. Fig. 5A,B), this was not related to enrichment for a specific subclass of the spliceosome (Suppl. Fig. 5C,D). For the remaining of the study, only the 61 and 24 overlapping factors will be used defining cluster 1 and 2 splicing factors, respectively.

### Association of co-regulated splicing factors with clinical breast cancer phenotypes

**Figure 2:**
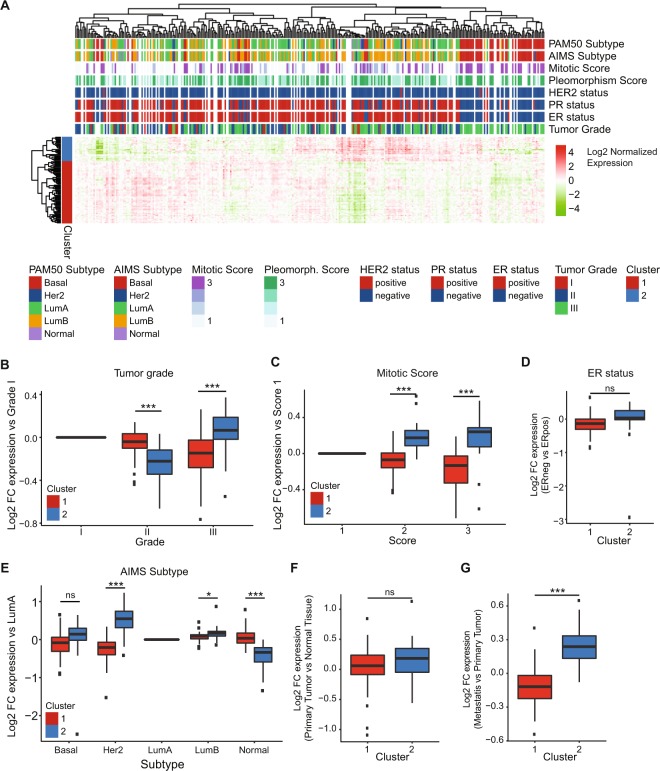
Association of co-regulated splicing factors with clinical breast cancer phenotypes. (**A**) Hierarchical clustering of cluster 1 and 2 SF RNA expression levels in BASIS RNA sequencing data. SF expression was log2 normalized, after which the for every SF the median was equalized to 0. (**B**) Cluster 1 and cluster 2 splicing factor expression levels in primary breast tumors with different tumor grades. SF expression compared to grade 1 tumors was calculated. (**C**) Cluster 1 and cluster 2 splicing factor expression levels in primary breast tumors with different mitotic scores. Fold changes compared to mitotic score 1 were calculated for all splicing factors. (**D**) Log2 fold change in expression of cluster 1 and cluster 2 splicing factors comparing ER negative to ER positive primary breast tumors. (**E**) Cluster 1 and cluster 2 splicing factor expression in AIMS breast cancer subtypes. (**F**) Log2 fold change in expression of cluster 1 and cluster 2 splicing factors comparing primary tumor to normal breast tissue. (**G**) Log2 fold change in expression of cluster 1 and cluster 2 splicing factors comparing primary tumor to metastatic tissue. Groups are compared using a student’s t-test. *P < 0.05, **P < 0.01, ***P < 0.001.

Next, we investigated whether the cluster 1 and cluster 2 splicing factors were related to a more aggressive breast cancer phenotype by examining clinical and pathological parameters such as tumor grading, mitotic score and survival. We performed an unsupervised clustering of median normalized log2 RNA expression levels of both groups of splicing factors in primary breast tumors derived from the BASIS dataset, as this dataset is rich in patient data. With just using the transcriptomic data of the identified 85 splicing factors belonging to either cluster 1 or 2, we could separate tumors based on clinical characteristics such as the basal PAM50 and AIMS subtype and hormone receptor status (Fig. 2A), but not on known driver gene mutations (Suppl. Fig. 6A). Then we compared RNA expression levels of our set of splicing factors between tumors with different pleomorphism scores, mitotic scores and tumor grading. Compared to well differentiated grade 1 tumors, moderately differentiated grade 2 tumors showed slightly decreased levels of cluster 2 splicing factors (Fig. 2B). However, poorly differentiated grade 3 tumors demonstrated the reverse effect exhibiting lower levels of cluster 1 factors, but higher cluster 2 factor levels compared to the low grade 1 tumors (Fig. 2B). Moreover, high pleomorphism and mitotic scores were associated with increased levels of cluster 2 factors and decreased levels of cluster 1 factors compared to low scored tumors (Fig. 2C, Suppl. Fig. 6D). Breast cancer invasiveness and progression is, amongst others, characterized by the subtype classification and hormone receptor status^14^. The estrogen receptor (ER) status has been demonstrated to be a favorable prognostic factor in breast cancer, especially in the first 5 years after diagnosis^15^. Confirming the observed tendency, cluster 1 splicing factors were slightly higher expressed in ER positive tumors, while cluster 2 factors were slightly higher expressed in the more aggressive ER negative tumors, yet both differences were not significant (Fig. 2D). This pattern was confirmed in the TCGA RNA sequencing dataset (Suppl. Fig. 6B,C). Moreover, compared to the less aggressive luminal A subtype, cluster 2 factors were higher expressed in the more aggressive luminal B, basal-like and HER2 amplified tumors while lower expressed in normal-like tumors. In contrary, cluster 1 factors were lower expressed in the more aggressive tumor types (Fig. 2E, Suppl. Fig. 6E). Altogether our data demonstrate that cluster 2 splicing factors are associated with a more aggressive tumor type which is in general related to a poorer prognosis.

Our analysis demonstrated that by comparing primary tumor tissue with either normal breast tissue or metastatic breast tissue, we did not observe differences in expression levels for all splicing factors comparing primary tumor tissue with either normal breast tissue or metastatic breast tissue (see Fig. 1A,E). Repeating this analysis for cluster 1 and 2 splicing factors independently also did not demonstrate a difference between primary tumor and normal breast tissue (Fig. 2F, Suppl. Fig. 7A). However, cluster 2 splicing factors were significantly overexpressed in metastatic tissue compared to primary tumor tissue (Fig. 2G, Suppl. Fig. 7B) suggesting an involvement of these factors in breast cancer metastasis formation.

**Figure 3:**
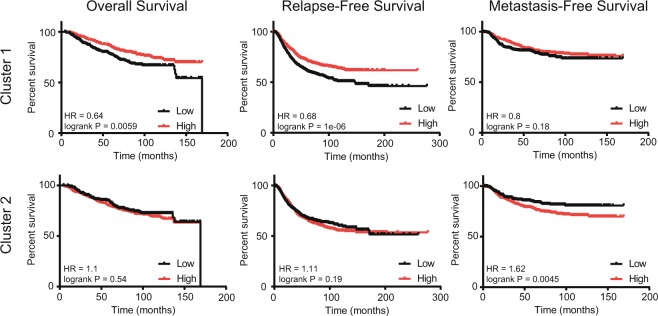
Association of the expression of cluster 1 and cluster 2 splicing factors with breast cancer overall survival. Per patient, mean expression of all factors within one cluster was calculated. Based on this mean expression, the patient cohort was median-split in low and high expression of cluster 1 or 2 splicing factors and survival curves for overall survival, relapse-free survival and metastasis-free survival were generated.

Furthermore, the relation of cluster 1 and 2 splicing factor expression levels to survival of breast cancer patients was examined. Here, we selected all breast cancer subtypes, calculated the mean expression of all cluster splicing factors and split the patient cohort by the median expression level. Interestingly, a high average expression level of cluster 1 factors is associated with increased overall and relapse-free survival (Fig. 3, Suppl. Fig. 8A). In contrast, a high average expression of cluster 2 factors is associated with decreased metastasis-free survival (Fig. 3, Suppl. Fig. 8A). We obtained similar results in ER negative tumors: high expression of cluster 1 factors was associated with prolonged overall survival, while high expression of cluster 2 factors was linked to decreased metastasis free survival (Suppl. Fig. 8B). In ER positive tumors, cluster 1 splicing factors do not show an association with overall survival, while high cluster 2 levels are associated with a less favorable relapse-free and metastasis-free survival (Suppl. Fig. 8C). Furthermore, also in the MA-867 dataset the Hazard Ratio (HR) for metastasis development was increased for cluster 2 splicing factors, especially in ER positive tumors (Suppl. Fig. 8D). In conclusion, we propose that cluster 2 splicing factors are predictors of both a poor relapse- and metastasis-free survival. We will further refer to these as breast cancer enhancing splicing factors (“Enhancer-SF”). Since cluster 1 splicing factors may act to suppress or delay the progression of breast cancer, we will further refer to these as breast cancer suppressing splicing factors (“Suppressor-SF”).

### Breast cancer Enhancer-splicing factors are linked to isoform specific gene expression

**Figure 4:**
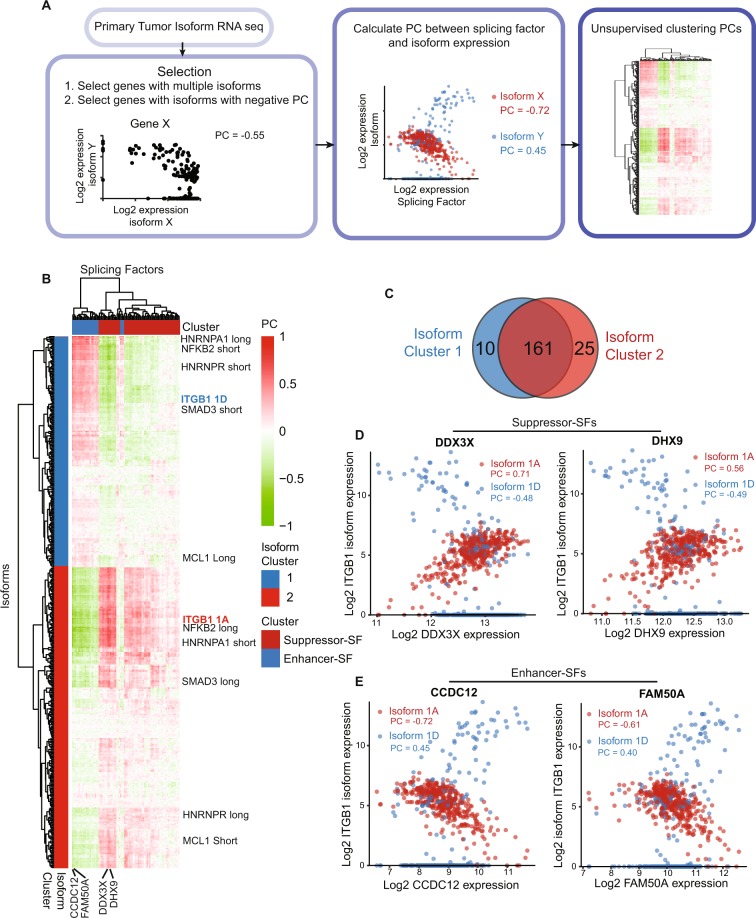
Linking breast cancer Enhancer-splicing factors to isoform specific gene expression. (**A**) Schematic overview of steps used to correlate Enhancer- and Suppressor-SF expression to isoform-specific gene expression. (**B**) Hierarchical clustering (Euclidean distance, complete linkage) of the PC of Enhancer- and Suppressor-SFs with isoform expression in TCGA RNA sequencing data (red = positive correlation, green = negative correlation). Based on this clustering the isoforms were divided in 2 clusters. (**C**) Number of genes in the isoform clusters. (**D**) Correlation between ITGB1 isoforms and Suppressor-SFs DDX3X and DHX9. (**E**) Correlation between ITGB1 isoforms and Enhancer-SFs CCDC12 and FAM50A.

Given that we could discriminate our co-regulated splicing factors in on the one hand breast cancer Enhancer-SF and the other hand breast cancer Suppressor-SF, we anticipated that the expression of these splicing factors would also be related to specific isoform expression patterns that might determine cancer progression. Therefore, we correlated the expression levels of Suppressor-SFs and Enhancer-SFs to gene isoforms using the TCGA isoform expression data. Here, we selected genes expressing multiple isoforms of which the expression levels of the individual isoforms are negatively correlated with each other (Fig. 4A). By performing the latter step, we selected genes that do switch isoforms comparing different primary tumors and remove isoforms that are only regulated on total gene expression level. Notably, breast tumor samples hardly expressed intermediate levels of both isoforms of selected genes resulting in one isoform being highly expressed while the other is absent, or high expression levels of both isoforms (Suppl. Fig. 9A). Hierarchical clustering of PCs between spliceosomal components and selected isoforms (Fig. 4B) allowed us to clearly discriminate between Suppressor-SFs and Enhancer-SFs, but also separated the isoforms in two clusters (Fig. 4B). Most of the selected genes had isoforms in both clusters (Fig. 4C, Suppl. Fig. 10) confirming that a switch in expression of splicing factors coincided with a downstream switch in splicing patterns. This could not be explained by a general increase in intron or exon inclusion, since the isoform length of isoforms in both clusters remained equal (Suppl. Fig. 9B). Interestingly, amongst the alternatively spliced genes were very well-known determinants of breast cancer progression, including integrin β1 (ITGB1). In primary human breast tumors, the most common ITGB1 isoform 1 A was positively correlated to Suppressor-SFs such as DDX3X and DHX9 (Fig. 4D) while being negatively correlated to Enhancer-SFs such as CCDC12 and FAM50A (Fig. 4E). ITGB1 isoform 1D includes an alternative exon resulting in a prolonged and distinct C-terminus and is known to be specifically expressed in muscle tissue^16,17^. Here, we identified increased expression of isoform 1D in primary breast tumors in relation to high expression levels of Enhancer-SFs (Fig. 4E). Furthermore, also SMAD3 (Suppl. Fig. 9C) and NFKB2 (Suppl. Fig. 9D) showed alternative splicing patterns when comparing Enhancer-SF FAM50A with Suppressor-SF DHX9. Interestingly, SMAD3 and NFKB2 are implicated in survival signaling by regulating MCL-1, which has two well-known isoforms^18^. The common long MCL-1 isoform which has pro-survival activity positively related to Enhancer-SFs; the short MCL-1 isoform which mediates apoptosis was correlated to Suppressor-SF levels (Suppl. Fig. 9E)^18^. Furthermore, we also noticed that splicing factors regulate their own splicing pattern as was observed for HNRNPA1 (Suppl. Fig. 9F). Although the implication for most of these alternative splicing patterns has to be elucidated, we can conclude that differential expression of Enhancer-SFs and Suppressor-SFs results in distinct isoform patterns for many genes, including genes that have been implicated in cancer progression.

### Breast cancer Enhancer-splicing factors are associated with the expression of mitochondrial electron transport, splicing, protein metabolism and decreased transcription

**Figure 5:**
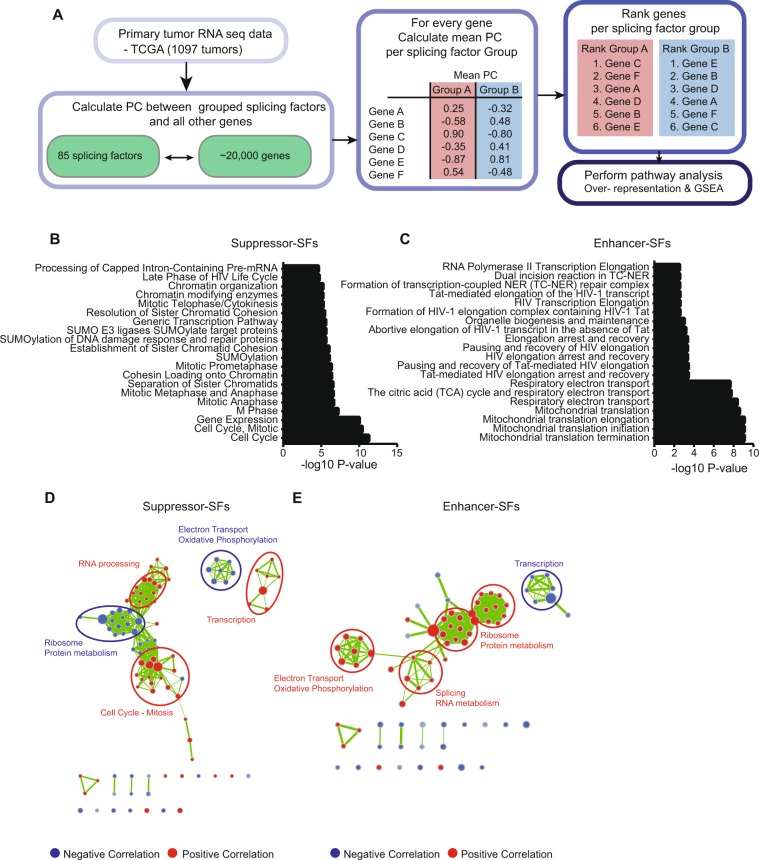
Association of Enhancer-SF expression levels with cancer-related biological programs. (**A**) Schematic overview of steps used to correlate Enhancer- and Suppressor-SF expression to other genes in the genome followed by over-representation and gene-set enrichment analyses. (**B**) Top altered pathways in over-representation analysis of the top 500 genes positively correlating with Suppressor-SFs. (**C**) Top altered pathways in over-representation analysis of the top 500 genes positively correlating with Enhancer-SFs. (**D**) Cytoscape visualization of GSEA of pathways highly correlated to Suppressor-SFs. (**E**) Same as in (**D**) but for Enhancer-SFs.

As a next step, we studied whether the expression of the Enhancer-SF genes was linked to specific signaling networks and/or biological programs. Therefore, we calculated the PC of the Enhancer-SFs and Suppressor-SFs to all the genes available in the TCGA RNA sequencing dataset, naturally excluding the splicing factors (Fig. 5A, Suppl. Table 4). Next, genes were ranked based on their mean correlation to Suppressor- and Enhancer-SFs, respectively. Over-representation analysis of the top 500 genes positively correlated with Suppressor-SFs were enriched in processes related to cell cycle and SUMOylation (Fig. 5B); top 500 genes that positively correlated with Enhancer-SFs were enriched in respiratory electron transport, mitochondrial translation, and also transcriptional elongation (Fig. 5C). Ranked Gene Set Enrichment Analysis (GSEA) confirmed the positive correlation of Suppressor-SFs with cell cycle, mitosis, RNA processing and transcription, while negatively correlating to genes linked with mitochondrial processes such as electron transport and oxidative phosphorylation, ribosomal pathways and protein metabolism (Fig. 5D). As expected, Enhancer-SFs show the opposite behavior, being positively related to mitochondrial respiration, splicing and protein metabolism and negatively related to transcriptional pathways (Fig. 5E). Since both analyses demonstrated an important relation of splicing factors to cell cycle, sister chromatid cohesion, mitosis (all positively related to Suppressor-SFs), respiratory electron transport and mitochondrial translation (positively related to Enhancer-SFs), we investigated these pathways in greater detail. We first focused on the cell cycle and mitosis related pathways and observed a dual behavior, meaning that the majority of genes in these pathways were positively related to Suppressor-SFs, while a substantial fraction displayed a negative relation (Suppl. Fig. 11). A further detailed assessment of these pathways revealed that Suppressor-SFs were mainly associated with genes implicated in negative cell cycle regulation, such as cell cycle checkpoints and arrest (Suppl. Fig. 11); these genes involved amongst others BRCA1, ATM, ATR and RAD genes, which are related to inhibition of cell cycle progression in reaction to defects in DNA replication or DNA damage.

Co-regulation of gene networks in cell biology is likely driven by transcription factors that bind similar promoter regions^19–22^. Therefore, we aimed to uncover the transcription factor networks that contribute to the expression of Enhancer- and Suppressor-SFs and their directly co-regulated gene expression patterns. To answer this question, we performed a binding motif enrichment analysis using transcription factor binding motifs from the Jaspar database combined with the Pscan algorithm^23^ for Enhancer- and Suppressor-SFs separately. Interestingly, binding sites of the transcription factors CAMP responsive element binding protein 1 (CREB1), cyclic AMP (cAMP) response element modulatory protein (CREM) and activating transcription factor 1 (ATF1) were highly enriched in Suppressor-SFs, while hardly present in Enhancer-SFs (Suppl. Fig. 12A). CREB1, ATF1, and CREM are members of a subfamily of the basic leucine zipper transcription factors that altogether can bind either as homo –or heterodimers to cAMP response elements located in the promoter regions of target genes^24^. We then selected i) the top 100 tumor samples expressing the highest level Suppressor-SFs and the lowest level of Enhancer-SFs (HS-LE tumors) and ii) the top 100 tumor samples expressing the lowest level of Suppressor-SFs and highest level of Enhancer-SFs (LS-HE tumors). Interestingly, the HS-LE tumors show significantly higher levels of ATF1 and CREB1 compared to LS-HE tumors, suggesting that these transcription factors may regulate the transcription of Suppressor-SFs (Suppl. Fig. 12B). CREM levels were equal in both tumor groups (Suppl. Fig. 12B). As expected, we verified that CREB1 and ATF1 levels were positively correlated to Suppressor-SF levels, while being negatively correlated with Enhancer-SF levels (Suppl. Fig. 12C). Altogether, our analysis suggests that ATF1/CREB1 transcription factors play an important role in regulating the expression of Suppressor-SF genes.

### Confirmation of genuine co-regulated expression of Enhancer-SF and Suppressor-SF gene sets in other cancer types

**Figure 6:**
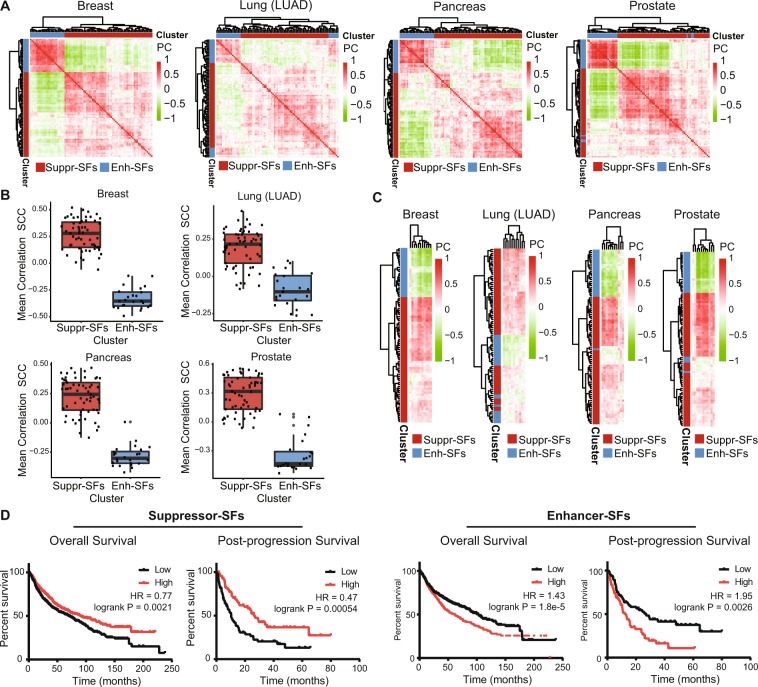
Enhancer-SF and Suppressor-SF expression levels are related to cancer progression in a wide variety of cancer types. (**A**) Hierarchical clustering (Euclidean distance, complete linkage) of Enhancer- and Suppressor-SF correlations in various cancer types. Red = highly positively correlated, green = highly negatively correlated. (**B**) Mean correlation of Suppressor- and Enhancer-SFs to the sister chromatid cohesion pathway in various cancer types. Here, for every SF, the correlation to the SCC pathway was calculated per patient, after which the mean patient correlation was calculated. (**C**) Hierarchical clustering (Euclidean distance, complete linkage) of PCs of Suppressor- and Enhancer-SFs to sister chromatid cohesion factors. (**D**) Per lung cancer patient, mean expression of all Suppressor- and Enhancer-SFs was calculated. Based on these expression levels, the patient cohort was median-split and overall and post-progression survival plots were generated^26^.

So far, we entirely focused on breast cancer due to the wealth of detailed datasets and tools available. Yet, we anticipated that the underlying alternative splicing through co-expression of splicing factors into similar Enhancer and Suppressor subgroups is a general phenomenon that would likely also be observed in other cancer types present in the TCGA database. We calculated the PC between all possible combinations of Enhancer- and Suppressor-SFs using RNA sequencing data for 32 different cancer types acquired from the TCGA database and performed unsupervised clustering on these datasets similar to the breast cancer dataset. We identified Enhancer- and Suppressor-SF gene expression correlations resembling the breast cancer dataset in almost all other cancer types, including common aggressive types such as lung, pancreas and prostate cancer, but not for testis and ovarian cancer (Fig. 6A, Suppl. Fig. 13). Importantly, also in other cancer types Enhancer-SF and Suppressor-SF expression were strongly correlated to genes involved in sister chromatid cohesion (SCC, Fig. 6B,C), activity of cell cycle, M-phase, mitochondrial translation and respiratory electron transport, similar as for the observations in breast cancer (Suppl. Fig. 14). Next, we examined the relation of Suppressor- and Enhancer-SFs to survival of lung and ovarian cancer patients^25,26^. In lung cancer patients, high expression levels of Suppressor-SF genes were significantly associated with prolonged overall survival as well as post-progression survival, while high expression of Enhancer-SF genes correlated significantly with shorter overall and post-progression survival in lung cancer patients (Fig. 6D). This is in contrast to ovarian cancer patients, that did not exhibit the strong negative correlation between Enhancer- and Suppressor-SF expression levels and consequently, did lack the correlation regarding patient survival (Suppl. Fig. 15).

## Discussion

RNA splicing is a complicated multistep process essential in multiple diseases amongst which cardiac disease, muscular dystrophy^4^ and cancer^6^. Common practice has been to define the role of single splicing factors in disease states^27,28^. Here, we took a bioinformatics approach to unravel splicing factor interactions at RNA expression levels in the context of cancer progression making advantage of the wealth of information provided by the TCGA and BASIS databases. By calculating PCs between the expression of all possible combinations of splicing factors in two independent human primary breast tumor RNA sequencing datasets, we identified two subgroups of splicing factors that are differentially co-expressed. One group of co-regulated splicing factors is particularly highly expressed in a more aggressive cancer phenotype and associated with poor survival rates, suggesting that the co-regulated expression of these splicing factors as a group contributes to cancer progression; we refer to this group as Enhancer-SFs of breast cancer. The other group of co-regulated splicing factors, which we refer to as Suppressor-SFs of breast cancer, are higher expressed in tumors with a more favorable prognosis. Importantly, we identified that these differential expression levels are associated with completely different alternative splicing patterns. In addition, expression of Enhancer-SF genes are negatively related to genes involved in negative cell cycle control and positively related to genes involved in mitochondrial respiration. We suggest that the poor clinical outcome of these high Enhancer-SF low Suppressor-SF patients might be caused by the combination of alternative splicing patterns, but also due to low expression levels of DNA damage repair genes as well as high levels of genes involved in oxidative phosphorylation. Importantly, we demonstrate that these relationships between splicing factors, pathways and survival outcome are not specific for breast cancer but are evident to most other cancer types, suggesting that the observed splicing factor co-regulation is a genuinely important driver in cancer progression.

At this stage, we cannot assign the causality of splicing factor subgroup co-expression in relation to cancer signaling networks and therefore it is too early to envision the implementation of the SF expression levels in clinical decision making. For example, we found that high expression of Enhancer-SFs in breast cancer is associated with high expression of genes involved in respiratory electron transport and mitochondrial translation, as well as a more aggressive clinical phenotype. The rewiring of cellular metabolism in order to promote survival of cancer cells is a well-known phenomenon^29^. The most profound metabolic adjustment in cancer cells is the increased activity of the less efficient glycolysis pathway over the more efficient oxidative phosphorylation, also known as the Warburg effect^30^. However, latest results suggest that oxidative phosphorylation is not completely lost during tumor progression and might even be important in metastasis formation^31^. Metastatic breast cancer cells exhibit elevated activity of mitochondrial respiration and patients bearing tumors with high levels of oxidative phosphorylation show a poorer clinical outcome^32,33^. From our observations, we cannot conclude that the differential expression of Enhancer-SFs is causing metabolic rewiring of cancer cells. Alternatively, changed Enhancer-SFs levels could be an effect instead of the cause of the altered activity in mitochondrial respiration. This could then indicate that the altered mitochondrial activity causes cells to switch their splicing pattern and subsequently, that metabolic rewiring and altered splicing patterns are both necessary to drive aggressiveness and poor clinical outcome. Further studies have to elucidate the causal link between splicing factors and the broad cellular transcriptional reprogramming to identify potential new targets for treatment or decide on a currently applied treatment strategy. Here, it would be interesting to start investigating the transcription factors ATF1 and CREB1, since these are potential transcriptional activators of the splicing factor clusters.

In this study we mainly focused on the correlation of RNA expression of Enhancer- and Suppressor-SFs to cancer signaling networks. Validating these correlations at protein levels would be essential to confirm the direct role of these SFs in tumor progression. As a first attempt, we calculated Spearman correlation coefficients to quantify the RNA and protein levels of 32 splicing factors in 77 primary tumors^34^ (Suppl. Fig. 16). The average correlation coefficient of the assessed splicing factors (cor = 0.26) resembled the top RNA-protein correlations of ten cancer types observed by Kosti *et al*.^35^. We did not always observe a strong correlation between RNA and protein expression levels of our splicing factors, suggesting that for some of these splicing factors, post-transcriptional processes can also be important for protein regulation. Although these splicing factors might be less involved in mediating downstream alternative splicing events, their RNA expression levels can still be used as a biomarker for the broad transcriptional reprogramming and metastatic behavior associated with the splicing factor levels. Further work would be needed to systematically link RNA expression to protein levels and next test the functional role of the Enhancer-SF in modulating particular programs that drive cancer progression.

The splicing factors act functionally in several large well-defined specific spliceosome complexes^3^. Intriguingly, neither Enhancer-SFs nor Suppressor-SFs could be assigned to specific spliceosome complexes. This observation underscores the complexity when investigating the role of single factors in such big complexes such as the spliceosome in relation to cancer progression. Likely the entire pattern of different factors, e.g. Enhancer-SF, is regulating the overall activity and functionality of the spliceosome and, thereby, driving defined RNA processing programs that drive tumor cell biology and cancer progression. For example, the differential increased expression of Enhancer-SF genes is associated with differential splicing of several genes including ITGB1. In primary human breast tumors the most common ITGB1 isoform A1 was negatively correlated to Enhancer-SF genes, yet positively correlated to Suppressor-SF genes. ITGB1 isoform 1D includes an alternative exon resulting in a prolonged and distinct C-terminus and is known to be specifically expressed in muscle tissue^16,17^. In our hands, the expression of the ITGB1 isoform 1D in primary breast tumors was particular related to high expression of Enhancer-SFs. While ITGB1 is a critical promotor of breast cancer progression^36,37^, in particular isoform 1D is known to affect focal adhesion kinase (FAK) and mitogen-activated protein kinase (MAPK) activation^38^, both known for their prominent role in (breast) cancer progression and metastasis formation^39,40^. This supports the rationale that the co-regulated Enhancer-SF genes drive RNA splicing leading to the formation of isoforms that are more prominently involved in aggressive cancer cell phenotypes.

Our data also indicates that the differential co-regulation of Enhancer-SFs and Suppressor-SFs is not specific for breast cancer, as we observed this phenomenon in most cancer types. For some even stronger differential expression than in the breast cancer cohort was observed, i.e. eye cancer and prostate cancer. Enhancer-SFs were also associated with poor prognosis in lung cancer. We hypothesize that several critical drivers of cancer progression that are active in different cancer types may determine the expression of either Enhancer-SFs or Suppressor-SFs, potentially by modulating the activity of CREB and ATF1 activity. Further studies need to await the unraveling of the activity of Enhancer-SFs.

In conclusion, our current analyses demonstrate the differential co-regulated expression of a subset of splicing factors that are associated with cancer progression. While this work sheds light on the role of the differential regulation of the spliceosome in cancer, differential expression of splicing factors might also drive the progression of other complex diseases. Furthermore, our bioinformatics approach may also be applied to the uncovering of the differential co-regulated expression of individual components that act in other large protein complexes that are critical in cancer progression, such as the translational machinery, respiratory or cell cycle systems or cell matrix adhesion complexes. This might illuminate unknown interactions, increasing the global understanding of these diseases and thereby contribute to the development of strategies to cure patients.

## Materials and Methods

### Data retrieval and normalization

RNA sequencing data from The Cancer Genome Atlas (TCGA) were obtained by using the TCGA Assembler R package^41^ after the new release in January 2017. Both normalized total gene expression and isoform-gene specific expression were extracted from this dataset. Normalized reads were log2 transformed before further analyses were performed. For correlation analysis, only solid primary tumor tissue samples were used. Tissue types and number of samples are shown in Suppl. Table 5.

RNA sequencing data as well as clinical data of a total cohort of 354 patients from the BASIS cohort were previously published and are publicly available^11^. Log2 normalized read counts were retrieved from the public repository.

Microarray data of primary tumors of 867 untreated patients (MA-867 dataset) was previously published and publicly available (GSE2034, GSE5327, GSE2990, GSE7390 and GSE11121).

All data used in manuscript is publicly available.

### Kaplan Meier survival curves

Kaplan Meier (KM) survival curves were obtained by using KM plotter with the multiclassifier function calculating the mean expression of the selected probes^42^. Patients were split by median expression levels. Overall, relapse-free and distant-metastasis-free survival curves were obtained for all breast cancer subtypes combined, but also for estrogen receptor positive and negative breast cancer subtypes separately.

### Hierarchical clustering

#### Correlation clustering

For the splicing factor correlation clusterings (Figs 1J,K, 6A, Suppl. Figs 2, 13), gene expression data of splicing factors were extracted from the RNA sequencing datasets. Pearson correlation coefficients (PCs) were calculated for all possible combinations of splicing factors. PCs were clustered using unsupervised clustering based on Euclidean distance and complete linkage. A cIValid stability test^43^ was performed to determine the optimal number of clusters for the primary correlation TCGA clustering (Fig. 1J), using a cluster number between 2 and 6 clusters as input. 4 clusters were selected based on the highest stability and major group representation. For the validation using the BASIS RNA sequencing data, an equal cluster number was used.

For isoform-splicing factor clusterings (Fig. 4B, Suppl. Fig. 10) and splicing factor-other gene clusterings (Fig. 6C, Suppl. Figs 11, 14 the same method was used but now PCs were calculated for isoform-splicing factor or splicing factor-gene combinations respectively.

#### Clinical Clustering

For the hierarchical clustering (Fig. 2A, Suppl. Fig. 6) linked to clinical data, log2 RNA expression values were median normalized per splicing factor across all patients. Normalized expression values were subjected to unsupervised clustering based on correlation and complete linkage.

### Expression levels in normal, primary tumor and metastatic tissue

Log2 fold change in expression levels of splicing factors (Suppl. Tables 1 and 2, Fig. 1A,E) were calculated using RNA sequencing data derived from the TCGA database (114 normal tissue samples and 7 metastatic tissue samples with matched primary tumor samples) using the following steps: (1) Calculation of the log2 fold change of splicing factor expression level between tumor and normal tissue (Suppl. Table 1) or tumor and metastatic tissue (Suppl. Table 2) per patient; tissue samples of the same patients were matched, (2) Calculation of the mean log2 fold change by taking the mean of all patients and (3) Significance determination by using a paired t-test. Adjusted p-value were calculated using the Benjamini-Hochberg correction for multiple testing. Since these methods were not based on a generalized linear model (GLM), the log2 fold changes of our initial method were compared to log2 fold changes calculated from raw RNA sequencing counts and the DESeq2 R packages that uses built in GLMs for normalization^44^. We observed a very strong and significant correlation between the log2 fold changes calculated with these two methods (Suppl. Fig. 17), suggesting that also the both methods are robust and reliable.

### Pathway analysis

To calculate the correlation between splicing factor expression levels and expression of other genes, PCs of Enhancer- and Suppressor-SFs with all other genes in the TCGA RNA sequencing data were calculated. For every gene, the average PC with Enhancer- and Suppressor-SFs was calculated. Based on PC, genes were ranked and subjected to pathway analysis.

#### Over-representation analysis

The top 500 correlated genes with Enhancer- or Suppressor-SFs were used for over-representation analysis. Over-representation analysis was performed using ConsensusPathDb^45^ using the Reactome pathway database.

#### Gene Set Enrichment Analysis (GSEA)

Ranked GSEA was performed on the full ranked gene lists^46^. Results were visualized using the GSEA plugin in Cytoscape version 3.4.0.

### Transcription factor analysis

Enrichment for transcription factor binding motifs in splicing factor start site regions was performed using the Pscan algorithm^23^ for Enhancer- and Suppressor-SFs separately. Enrichment was determined −50–450 nucleotides from the splicing factor start site. All transcription factors in the JASPAR transcription factor database were included in the analysis.

### Statistical analysis

Data were compared with Student’s t-test (two-tailed, equal variances) or one-way ANOVA (for comparison of more than 2 groups) using GraphPad Prism 6.0.

## Supplementary information

Supplementary figures and legends

Supplemental Table 1

Supplemental Table 2

Supplemental Table 3

Supplemental Table 4

Supplemental Table 5
