# Supplementary material for: Co-regulated gene expression of splicing factors as drivers of cancer progression
Source: Sci Rep. 2019 Apr 2;9:5484. doi: 10.1038/s41598-019-40759-4 (PMC6445126; doi:10.1038/s41598-019-40759-4)

Co-regulated gene expression of splicing factors as drivers of cancer progression

Esmee Koedoot[1], Marcel Smid[2], John A. Foekens[2], John W.M. Martens[2], Sylvia E. Le Dévédec[1], Bob van de Water[1,3]

[1] Division of Drug Discovery and Safety, LACDR, Leiden University, the Netherlands

[2] Department of Medical Oncology and Cancer Genomics Netherlands, Erasmus MC Cancer Institute, Erasmus University Medical Center, Rotterdam, the Netherlands

[3] To whom correspondence should be addressed

[3]Address of correspondence:

Bob van de Water
Division of Drug Discovery and Safety
Leiden Academic Centre for Drug Research
Leiden University
2300 RA Leiden
The Netherlands
Tel: +31-71-5276223
Fax: +31-71-5274277
b.water@lacdr.leidenuniv.nl

Running Title: Co-regulated expression of splicing factors in cancer

Supplementary Figure Legends

Supplementary Figure 1. Change in splicing factor RNA expression levels comparing normal, tumor tissue and metastatic tissue. **A)** Unsupervised clustering of log2 fold change in splicing factor expression levels comparing tumor to normal tissue (n = 116, Euclidean distance, complete linkage). **B)** Unsupervised clustering of log2 fold change in splicing factor expression levels comparing tumor to metastatic tissue (n = 7, Euclidean distance, complete linkage).

Supplementary Figure 2. Unsupervised clustering of PC of splicing factor RNA expression levels. **A)** Unsupervised clustering (Euclidean distance, complete linkage) of the PC of splicing factor RNA expression levels based on TCGA RNA sequencing data as in Fig. 1J, displaying all splicing factors. **B)** Unsupervised clustering (Euclidean distance, complete linkage) of the PC of splicing factor RNA expression levels based on BASIS RNA sequencing data as in Fig. 1K, displaying all splicing factors.

Supplemental Figure 3. TCGA cluster correlations. **A)** Hierarchical clustering (Euclidean distance, complete linkage) of the correlation of splicing factor expression levels in TCGA RNA sequencing data (red = high positive correlation, green = high negative correlation). Similar clustering as in Figure 1J, but now with all clusters numbered. **(B-G)** Hierarchical clustering (Euclidean distance, complete linkage) of the correlation of splicing factor expression levels of all cluster combinations shown in figure A.

Supplementary Figure 4. Pearson Correlation of RNA expression levels of cluster 1 and cluster 2 splicing factors in 867 primary breast tumors of untreated patients (MA-867).

Supplementary Figure 5. Distribution of cluster 1 and 2 splicing factors over different spliceosome sub-complexes. **A)** Cluster 1 splicing factor distribution over core and non-core categories. **B)** Same as in A but now for Cluster 2 splicing factors. **C)** Cluster 1 splicing factor

distribution over different spliceosomal sub-complexes. **D)** Same as in C, but now for Cluster 2 splicing factors.

Supplementary Figure 6. High cluster 2 splicing factor expression levels are related to a more aggressive breast tumor phenotype. **A)** Unsupervised clustering SF RNA expression in primary breast tumors of BASIS RNA sequencing data, annotated with known driver gene mutations. SF levels were first log2 normalized. Next median patient levels were equalized to 0 per SF. **B)** Log2 fold change in expression of cluster 1 and cluster 2 splicing factors comparing ER negative to ER positive primary breast tumors using the TCGA RNA sequencing dataset **C)** Hierarchical clustering of SF expression levels in primary breast tumors of TCGA RNA sequencing data. SF levels were first log2 normalized, followed by equalizing median patient levels to 0 per SF. **D)** Cluster 1 and cluster 2 splicing factor expression levels in primary breast tumors with different pleomorphism scores. Per SF, fold changes were calculated compared to score 1. **E)** Cluster 1 and cluster 2 splicing factor expression in PAM50 breast cancer subtypes. Groups are compared using a student's t-test. * $P<0.05$, ** $P<0.01$, ***$P<0.001$.

Supplementary Figure 7. Log2 difference in cluster 1 and 2 expression levels comparing **A)** primary tumor tissue with normal tissue and **B)** primary tumor tissue with metastatic tissue. Dots represent different patients.

Supplementary Figure 8. **A)** Association of the expression of cluster 1 and cluster 2 splicing factors with breast cancer overall survival. Per patient, mean expression of all factors within one cluster was calculated. Based on this mean expression, the patient cohort was median-split in low and high expression of cluster 1 or 2 splicing factors and survival curves for overall survival, relapse-free survival and metastasis-free survival were generated. Breast cancer survival curves of cluster 1 and cluster 2 splicing factors in estrogen receptor (ER) negative **(B)** and ER positive **(C)** subtypes. Per patient, mean expression of all factors within

one cluster was calculated. Based on this mean expression, the patient cohort was median-split in low and high expression of cluster 1 or 2 splicing factors and survival curves for overall survival, relapse-free survival and metastasis-free survival were generated. **D)** Hazard ratio (HR) for distant metastasis formation for cluster 1 and cluster 2 splicing factors in all (left) and estrogen receptor (ER) positive (right) tumors in the MA-867 dataset.

Supplementary Figure 9. **A)** Examples of expression levels of multiple isoforms of one gene in human breast cancer patient primary tumor RNA sequencing data. **B)** Density plot of difference in isoform length comparing cluster 1 and cluster 2 isoforms of the same gene. The length of the isoform in cluster 2 was subtracted from the length of the same gene isoform in cluster 1. Isoform clusters are based on Figure 4B. **C)** PCs of SMAD3 isoforms with Suppressor-SF DHX9 and Enhancer-SF FAM50A. **D)** Same as in C, but for NFKB2 isoforms. **E)** Same as in C, but for MCL1 isoforms. **F)** Same as in C, but for HNRNPA1 isoforms.

Supplementary Figure 10. Zoom in of isoform splicing factor clustering in Fig. 4B.

Supplementary Figure 11. Unsupervised clustering (Euclidean distance, complete linkage) of PC of Suppressor- and Enhancer-SFs with genes known to be involved in different stages or regulatory pathways of cell cycle. Red = highly positively correlated, green = highly negatively correlated.

Supplementary Figure 12. Transcription factors ATF1 and CREB1 can bind promoter regions of Suppressor-SFs but not Enhancer-SFs. **A)** Transcription binding site enrichment for splicing factors in promoter regions of Enhancer- and Suppressor-SFs. **B)** Expression levels of ATF1, CREB1 and CREM in High-Suppressor Low-Enhancer and Low-Suppressor High-Enhancer primary breast tumors. **C)** Unsupervised clustering (Euclidean distance,

complete linkage) of PCs of CREB1, ATF1 and CREM with Suppressor- and Enhancer-SFs. Red = highly positively correlated, green = highly negatively correlated.

Supplementary Figure 13. Hierarchical clustering (Euclidean distance, complete linkage) of PCs of Suppressor- and Enhancer-SFs in different cancer types. Red = highly positively correlated, green = highly negatively correlated.

Supplementary Figure 14. Hierarchical clustering (Euclidean distance, complete linkage) of Suppressor- and Enhancer-SF PCs to genes involved in mitochondrial translation, cell cycle, M phase and respiratory electron transport in breast cancer, lung cancer, pancreas cancer and prostate cancer. Red = highly positively correlated, green = highly negatively correlated.

Supplementary Figure 15. Enhancer- and Suppressor-SF expression levels related to overall survival, post-progression survival and progression-free survival in ovarian cancer. Per ovarian cancer patient, mean expression of all Suppressor- and Enhancer-SFs was calculated. Based on these expression levels, the patient cohort was median-split and overall and post-progression survival plots were generated[25].

Supplementary Figure 16**.** RNA-protein spearman correlations of splicing factors. RNA sequencing data was derived from TCGA, protein expression data was derived from Mertins et al[34].

Supplementary Figure 17. Correlation of log2 fold change of splicing factor expression comparing normal to tumor tissue with 1) the non-GLM method using normalized counts data provided by the TCGA portal and 2) using raw sequencing counts and a GLM method.

Supplementary Figure 1

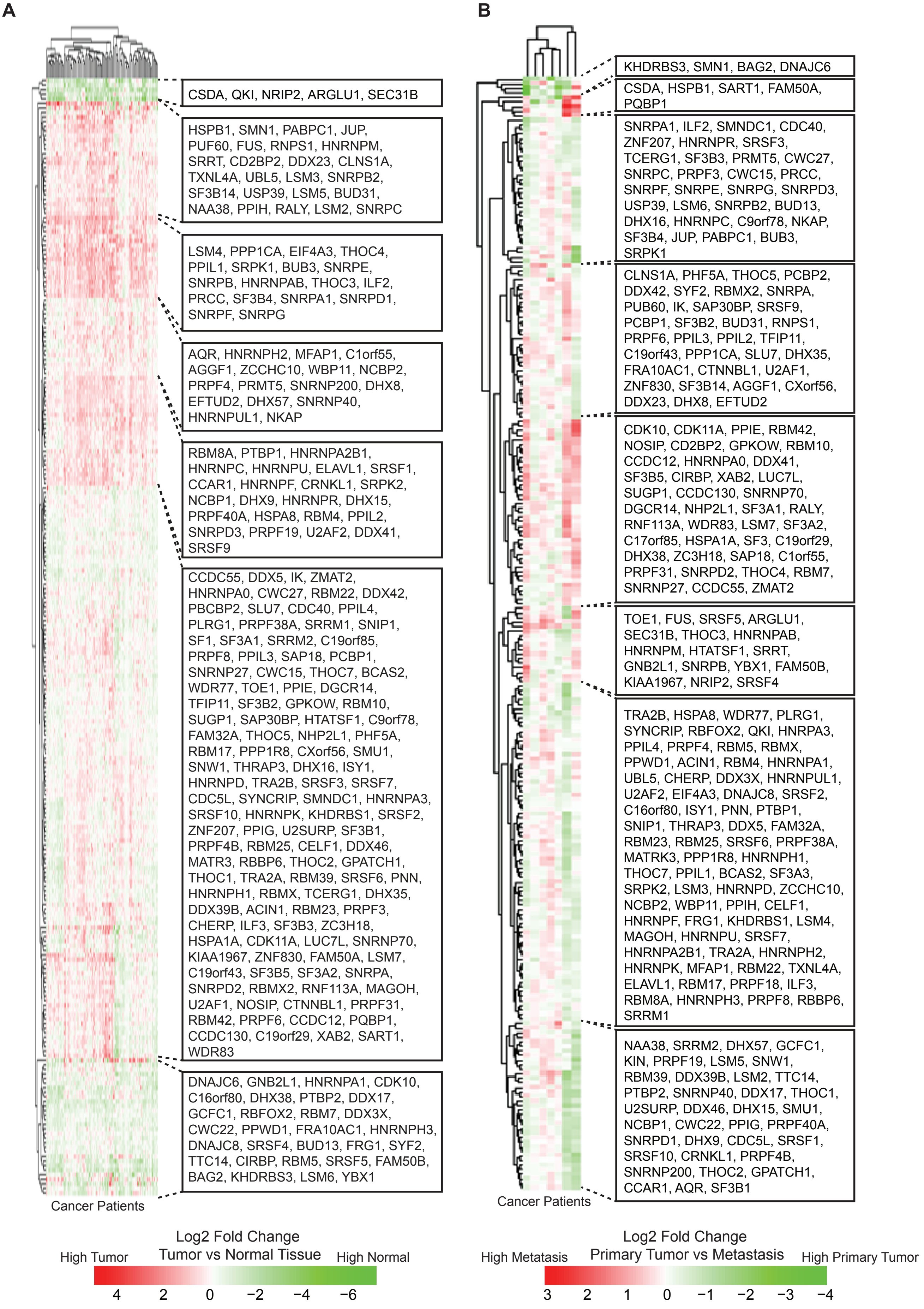

Supplementary Figure 2

A

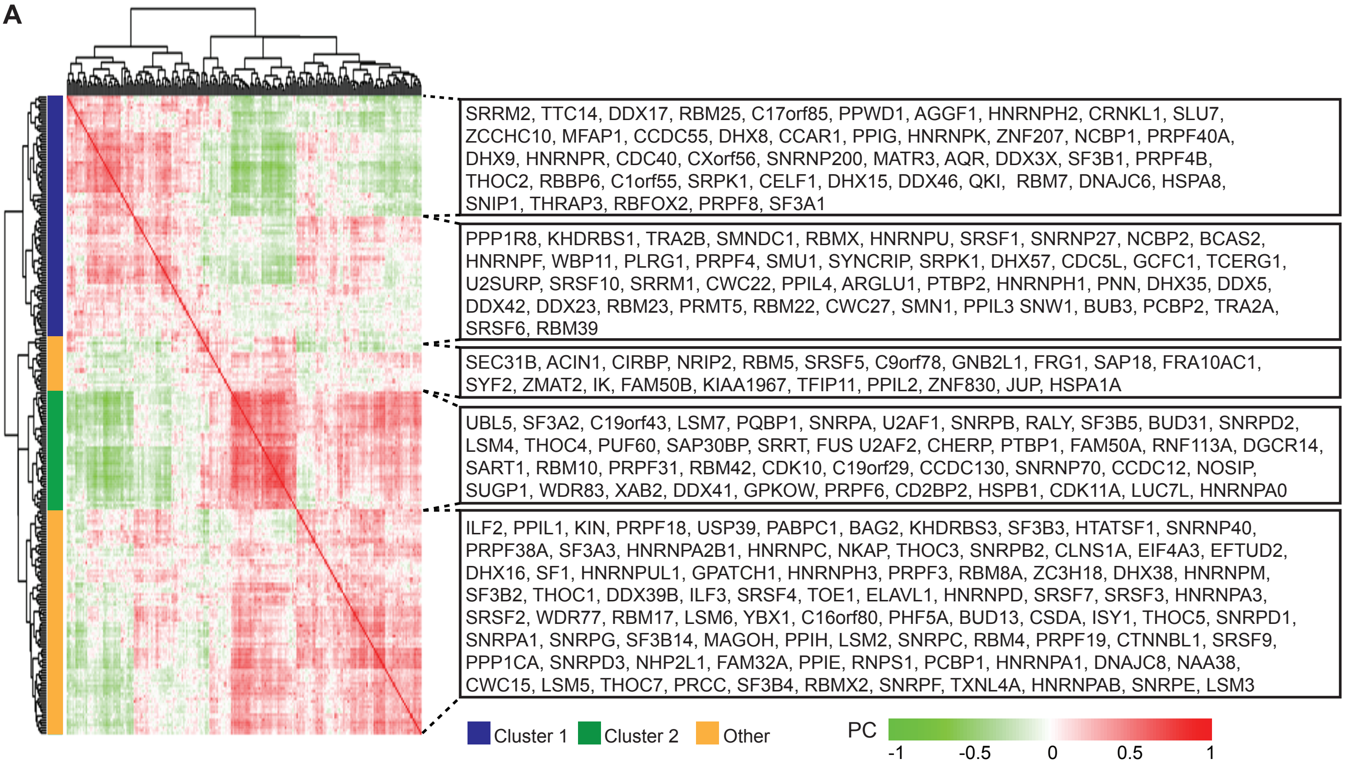

B

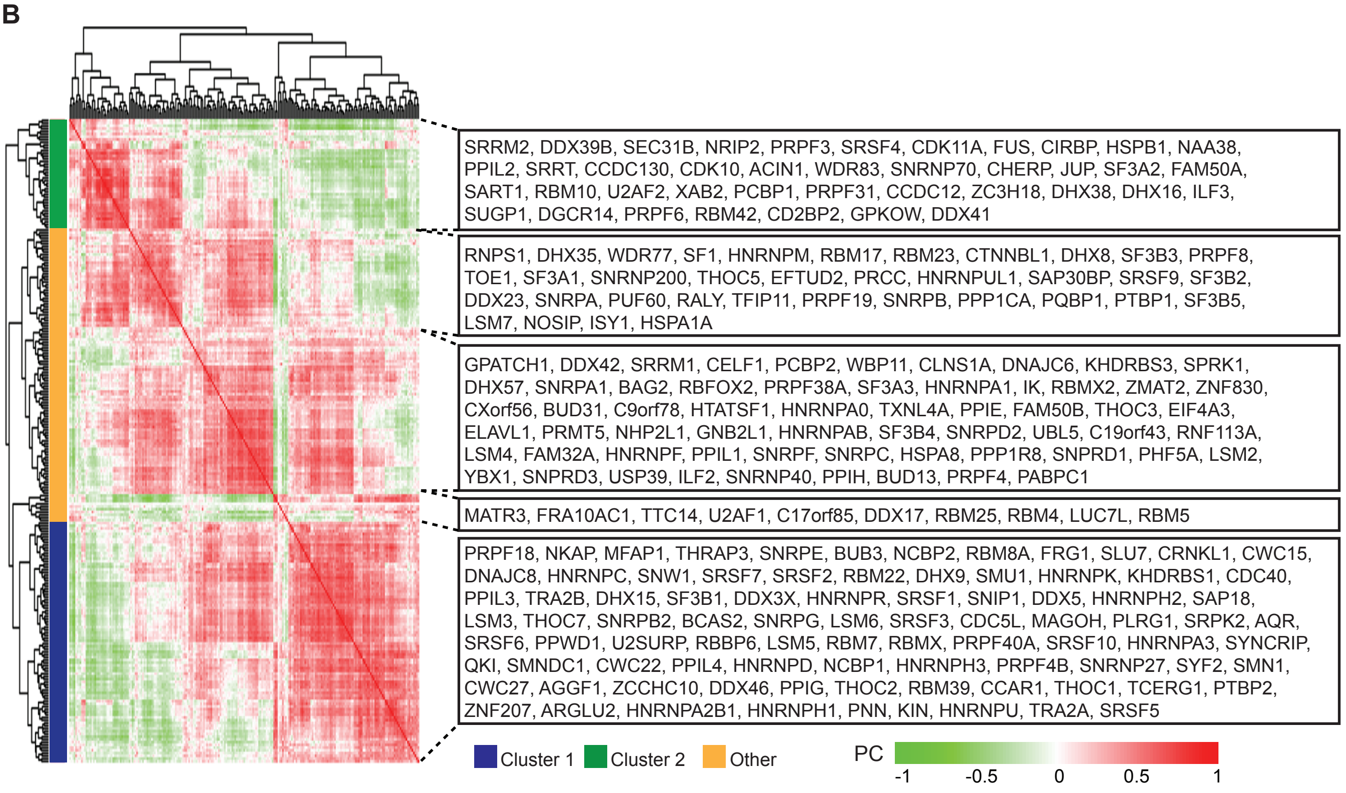

Supplementary Figure 3

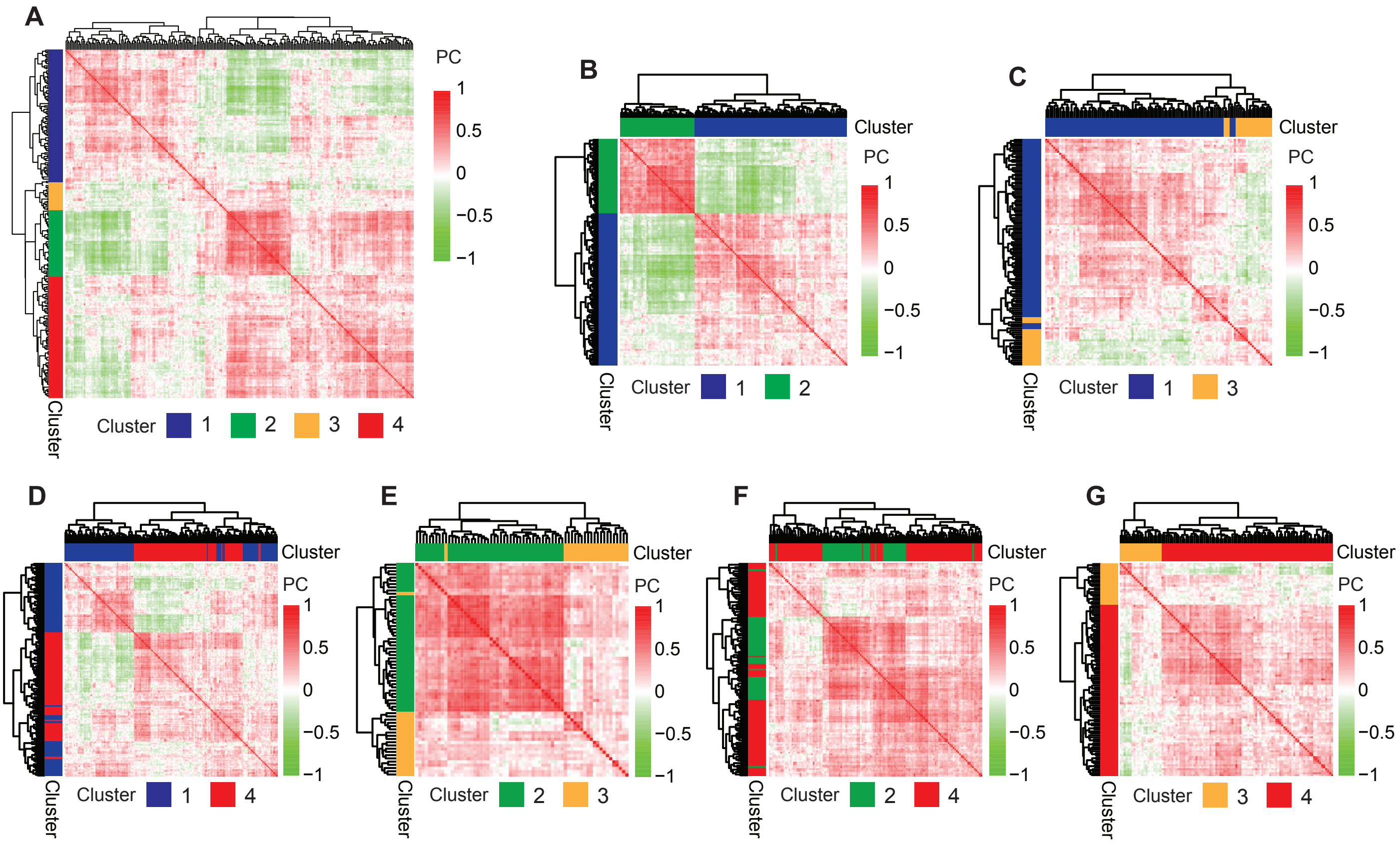

Supplementary Figure 4

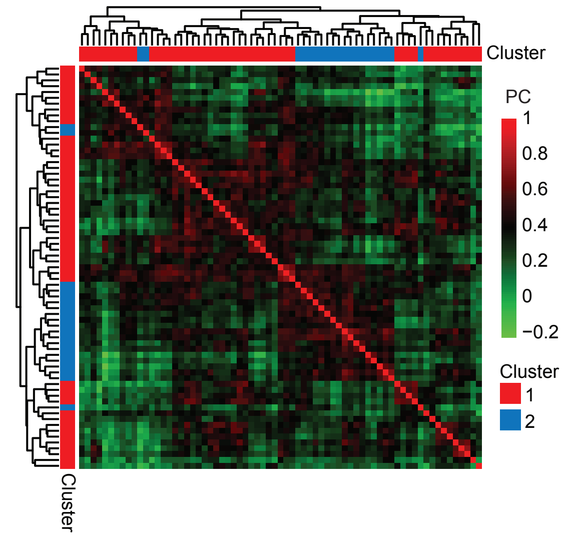

Supplementary Figure 5

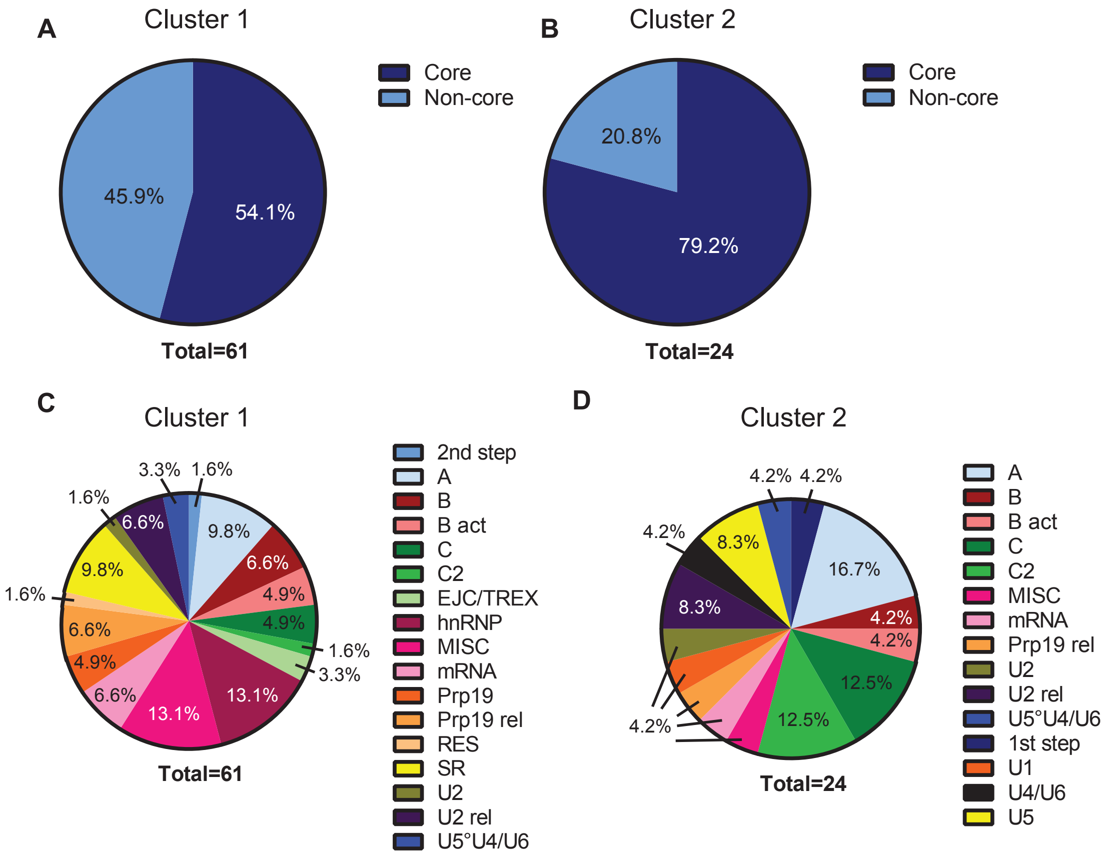

Supplementary Figure 6

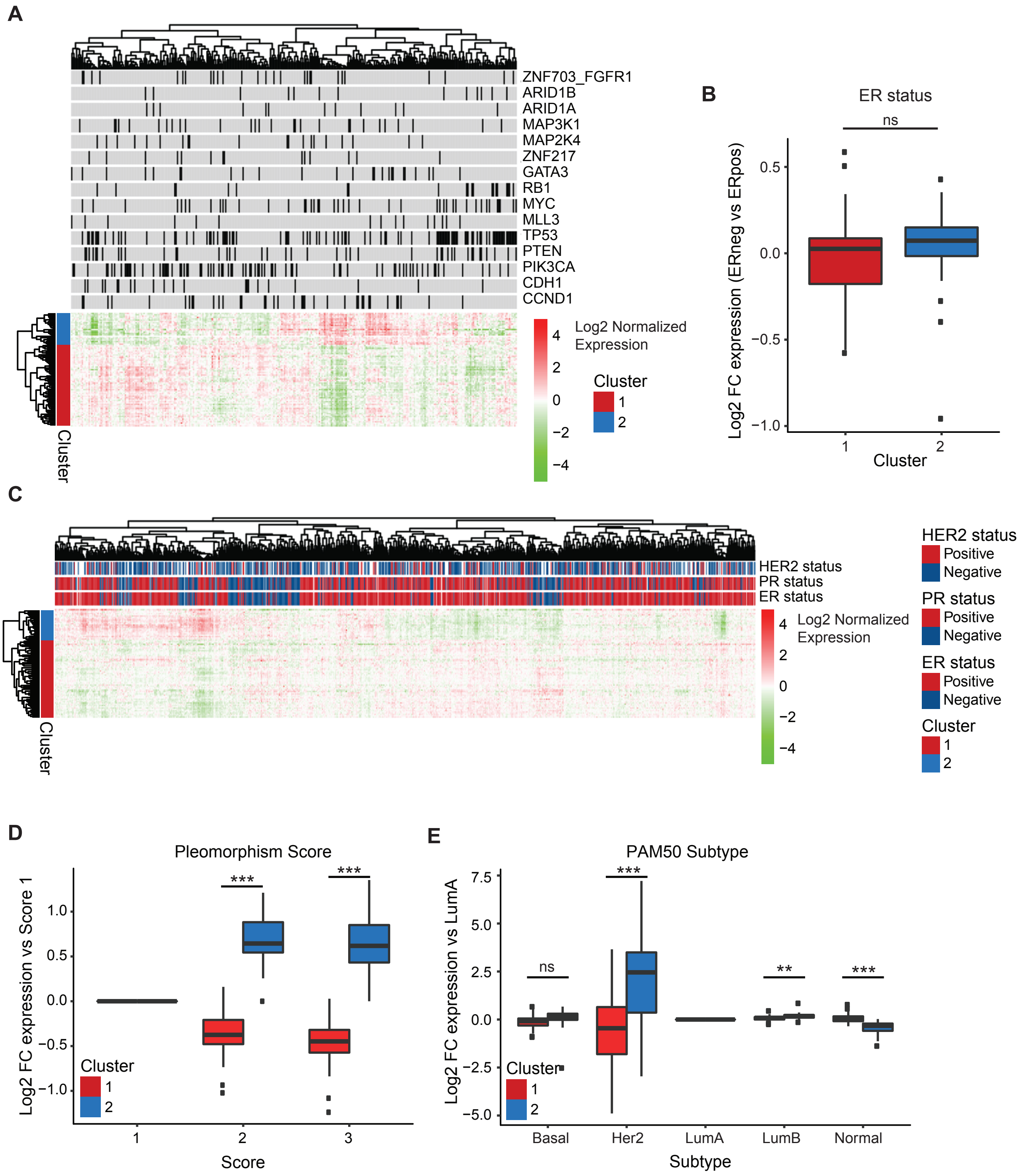

Supplementary Figure 7

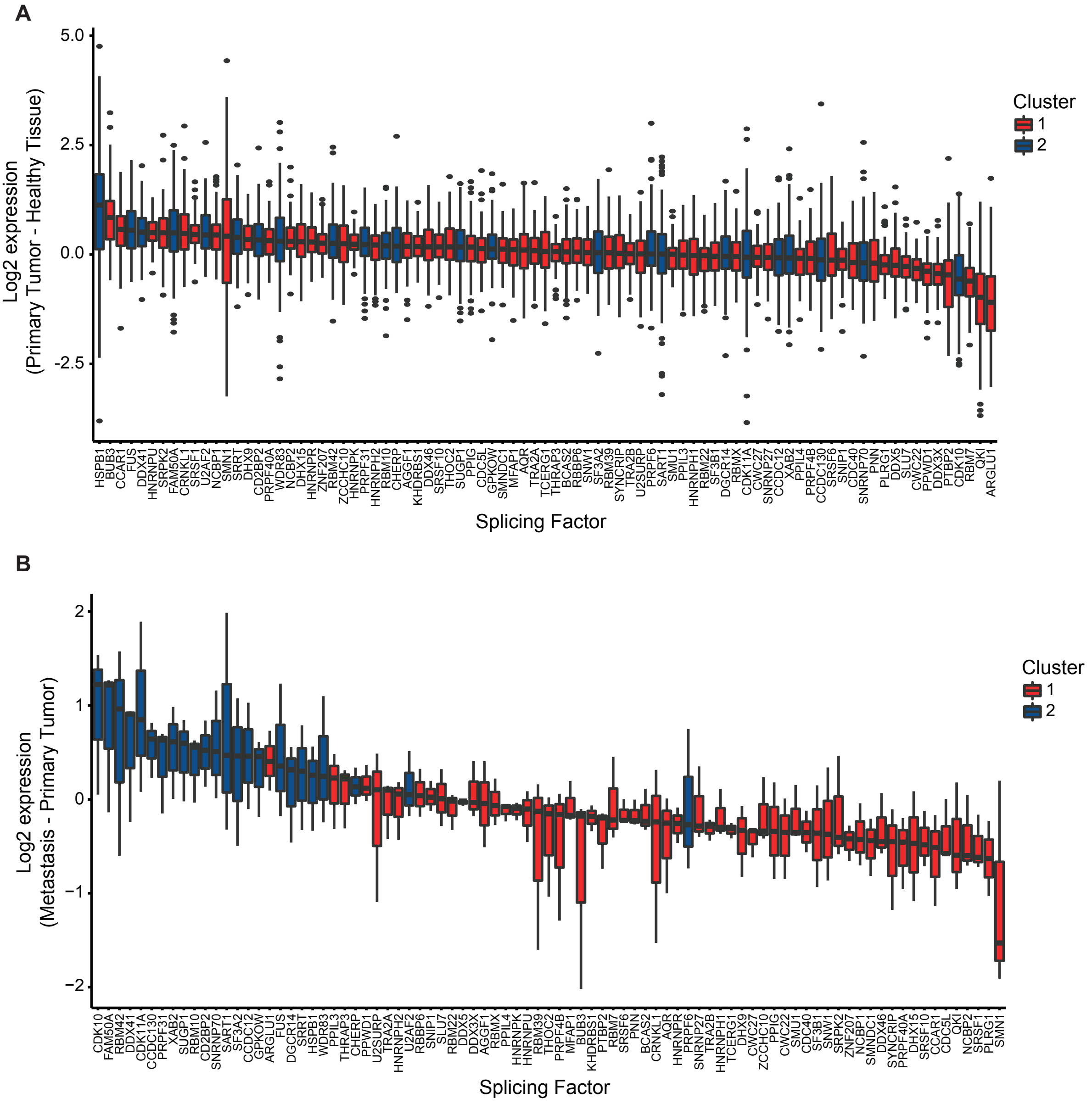

Supplementary Figure 8

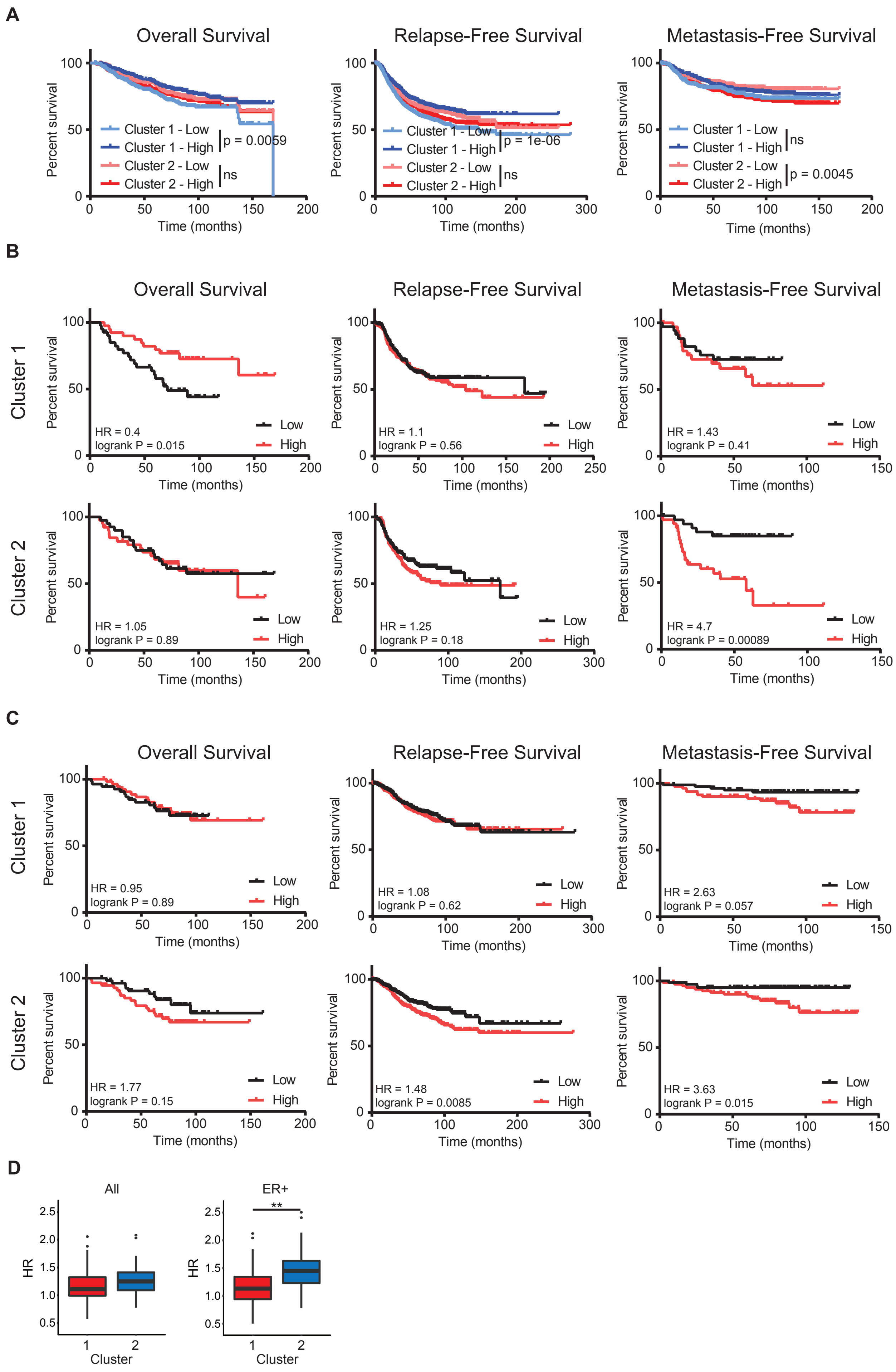

Supplementary Figure 9

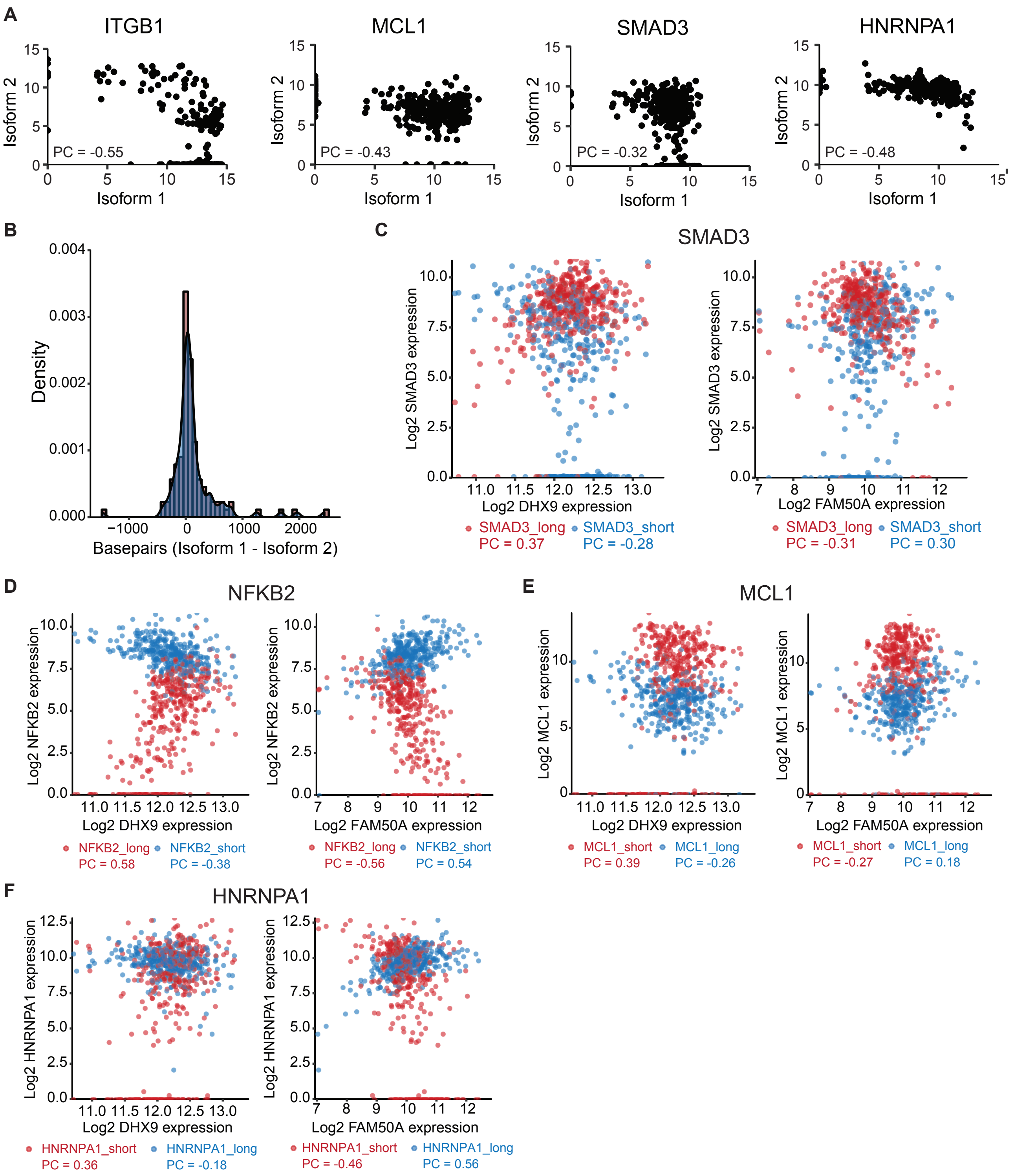

Supplementary Figure 10

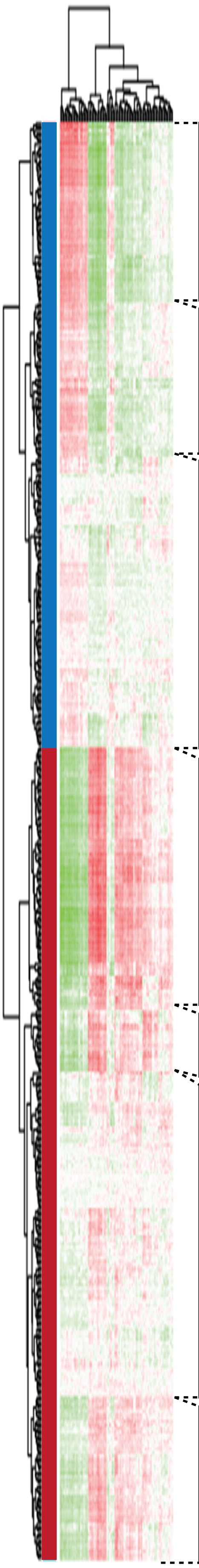

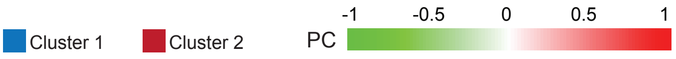

ARL6IP4-NM_001002252	NFIA-NM_005595	ITIH5-NM_032817	ADCY6-NM_020983	GRINL1A-NM_001018102
MINK1-NM_170663	MDM1-NM_017440	HNRNPR-NM_001102397	SEC61A2-NM_001142628	TDRKH-NM_001083964
HNRNPA1-NM_031157	NAT10-NM_001144030	ADPGK-NM_031284	NEDD1-NM_001135176	YME1L1-NM_014263
SCMH1-NM_001031694	PRKACB-NM_002731	RASAL2-NM_004841	DUT-NM_001025248	ITGB1-NM_033668
NFKB2-NM_001077494	HYOU1-NM_006389	GAS7-NM_201433	HK1-NM_033496	COPS2-NM_004236
KIAA0652-NM_001142673	ACSL5-NM_016234	GTF2H1-NM_005316	SMARCC2-NM_139067	
ZFYVE1-NM_178441	DNM1L-NM_012062	UACA-NM_018003	RAB5C-NM_004583	
ABI1-NM_001012752	PML-NM_033244	ZC3H14-NM_207661	IRF7-NM_004031	
WAC-NR_024557	SYTL2-NM_206929	EPS15-NM_001159969	AP3M1-NM_012095	
GLG1-NM_001145667	MIER1-NM_001077704	FBXO3-NM_012175	CHORDC1-NM_001144073	
INPP5K-NM_016532	DCAF8-NR_028105	RPS6KA1-NM_002953	MAX-NM_002382	
LAMB3-NM_001127641	RFX5-NM_001025603	WDR20-NM_181308	RNF6-NM_183043	

ETS1-NM_001162422	TXNDC16-NM_001160047	CYLD-NM_015247	TRIM34-NM_130389	STARD13-NM_178007
SMAD3-NM_001145104	RAB27A-NM_183236	CMIP-NM_030629	ZNF200-NM_198087	SPG20-NM_001142294
NCOA4-NM_001145261	NET1-NM_001047160	KIAA1598-NM_018330	PLEKHM1-NR_027774	MADD-NM_001135943
CARS-NM_139273	EIF2C3-NM_177422	SLC43A3-NM_017611	PC-NM_022172	ACD-NM_001082486
CYB561D1-NM_001134402	CSDE1-NM_001007553	LSP1-NM_002339	XRCC3-NM_005432	AK2-NM_013411
PCCA-NM_001127692	PTPRB-NM_002837	C11orf68-NM_001135635	S100PBP-NM_022753	MYL4-NM_002476
NCDN-NM_001014839	LBR-NM_194442	ACVRL1-NM_000020	FES-NM_002005	BRP44-NR_026550
TCTN3-NM_001143973	GLG1-NM_001145666	GMPR2-NM_001002000	CREBBP-NM_001079846	NBL1-NM_005380
HIC1-NM_001098202	SORBS1-NM_024991	TMOD2-NM_001142885	DZIP1-NM_014934	MPG-NM_002434

ZNF385A-NM_001130968	NECAP2-NM_001145278	C11orf68-NM_031450	CASC2-NR_026940	ABR-NM_021962
GSPT1-NM_001130007	TPM1-NM_001018020	NACA-NM_001113201	NACA-NM_001113203	DOCK9-NM_001130049
SIGIRR-NM_021805	NKIRAS2-NM_001001349	LINS1-NM_001040614	FOLR2-NM_001113536	MTMR15-NM_001146095
PLEKHG5-NM_020631	MYO19-NM_001033580	GON4L-NM_001037533	RPAIN-NM_001033002	TRAF5-NM_004619
C11orf73-NR_024597	SMCR7-NM_148886	AMN1-NR_004854	GALE-NM_001008216	TRAF5-NM_145759
LSP1-NM_001013255	SLC25A24-NM_213651	LQK1-NR_027286	UBTF-NM_014233	
CASP7-NM_033339	FTSJD1-NM_018348	APAF1-NM_013229	PKM2-NM_182470	
LRRC27-NM_030626	PRDM10-NM_199439	CDK11A-NM_024011	PLEKHG5-NM_001042663	
FAM186B-NR_027450	ATP6V0A1-NM_001130021	TPM3-NM_001043353	SF1-NM_201995	
USP44-NM_032147	LCK-NM_001042771	RNF41-NM_194359	CUL4A-NM_001008895	
MOBKL2C-NM_145279	C12orf72-NM_001135864	DDHD1-NM_001160147	ENTPD1-NM_001164178	
DIS3-NM_001128226	AMICA1-NM_153206	KBTBD4-NR_024222	C12orf65-NM_001143905	
CHEK1-NM_001114122	C10orf78-NM_001002759	CACNB1-NM_199248	ATP2A1-NM_004320	
C14orf159-NM_001102369	LGALS12-NM_001142536	ABCG4-NM_001142505	NKIRAS2-NM_001144927	
LRRC43-NM_001098519	LCMT1-NM_001032391	C15orf40-NM_001160114	AOC2-NM_009590	
LRRC27-NM_001143757	ASPA-NM_001128085	PHYHIPL-NM_001143774	C11orf73-NM_016401	
LPXN-NM_001143995	PTPN5-NM_001039970	CYP19A1-NM_031226	WNT5B-NM_030775	
OS9-NM_001017958	SH2B1-NM_001145795	THYN1-NM_014174	MCL1-NM_182763	
CNTN1-NM_175038	ABCG4-NM_022169	ZNF385A-NM_015481	NIN-NM_182946	
MYL4-NM_001002841	SLCO1C1-NM_001145944	SP1-NM_003109	TARBP2-NM_134323	

HYOU1-NM_001130991	NFIA-NM_001134673	CREBBP-NM_004380	S100PBP-NM_001017406	CHEK1-NM_001274
CMIP-NM_198390	ZFYVE1-NM_021260	UACA-NM_001008224	NAT10-NM_024662	CHORDC1-NM_012124
RNF6-NM_005977	ABR-NM_001092	WAC-NM_016628	PRDM10-NM_199437	DUT-NM_001948
ABI1-NM_005470	SORBS1-NM_015385	AP3M1-NM_207012	SP1-NM_138473	ADPGK-NR_023318
KIAA0652-NR_024589	FTSJD1-NM_001099642	MIER1-NM_001077701	APAF1-NM_181861	MYO19-NM_001163735
CARS-NM_001014438	PRKACB-NM_182948	COPS2-NM_001143887	TXNDC16-NM_020784	DZIP1-NM_198968
MADD-NM_130476	DOCK9-NM_001130050	ITGB1-NM_033666	KIAA1598-NM_001127211	TMOD2-NM_014548
NCDN-NM_014284	PTPRB-NM_001109754	RASAL2-NM_170692	TCTN3-NM_015631	SPG20-NM_001142295
MINK1-NM_153827	ETS1-NM_001143820	ITGB1-NM_002211	FBXO3-NM_033406	DIS3-NM_014953
KBTBD4-NM_018095	STARD13-NM_052851	NIN-NM_020921	GSPT1-NM_002094	
SCMH1-NM_012236	SEC61A2-NM_018144	CSDE1-NM_001130523	ZC3H14-NM_207660	
DCAF8-NR_028103	SYNE2-NM_015180	GON4L-NM_032292	ATP6V0A1-NM_001130020	
TDRKH-NM_006862	YME1L1-NM_139312	NCOA4-NM_005437	HNRNPA1-NM_002136	
RNF41-NM_194358	GTF2H1-NM_001142307	NFKB2-NM_002502	YY1AP1-NM_139118	
HK1-NM_000188	EIF2C3-NM_024852	GRINL1A-NM_015532	BRP44-NM_001143674	
MIER1-NM_020948	RNF41-NM_005785	EPS15-NM_001981	PKM2-NM_002654	
PLEKHM1-NM_014798	SLC25A24-NM_013386	NEDD1-NM_152905	LBR-NM_002296	

TARBP2-NM_134324	SMARCC2-NM_003075	MTMR15-NM_001146096	ADCY6-NM_015270	TRAF5-NM_001033910
SYTL2-NM_206928	MDM1-NM_020128	GMPR2-NM_016576	SYTL2-NM_001162953	PCCA-NM_000282
TRIM34-NM_021616	C12orf72-NM_001135863	AMN1-NM_001113402	SMAD3-NM_005902	
RAB5C-NM_201434	SYTL2-NM_206927	LINS1-NM_001040615	GPHN-NM_020806	

CHEK1-NM_001114121	PCBP2-NM_001128911	YY1AP1-NM_139119	MINK1-NM_015716	PRR5L-NM_001160167
SLC43A3-NM_014096	GPHN-NM_001024218	SMCR7-NM_139162	HTR7-NM_019860	RAB34-NM_001144942
OS9-NM_001017956	LQK1-NR_027285	HNRNPR-NM_001102399	CYP19A1-NM_000103	RUSC1-NM_001105203
GALE-NM_000403	CHD3-NM_001005273	SH2B1-NM_015503	GRM5-NM_001143831	SLC25A3-NM_002635
CACNB1-NM_199247	COQ10A-NM_144576	C12orf72-NM_173802	RPAIN-NM_001160243	FAM186B-NM_032130
LCK-NM_005356	MPG-NM_001015054	COQ10A-NM_001099337	GRM5-NM_000842	C1orf183-NM_198926
GFI1-NM_001127216	RAB34-NM_001144943	CASC2-NR_026939	AOC2-NM_001158	THYN1-NM_001037305
LAMB3-NM_000228	ENTPD1-NM_001164179	PCBP2-NM_005016	TDRKH-NM_001083963	
PHYHIPL-NM_032439	FOLR2-NM_001113534	KAZ-NM_001018001	SLCO1C1-NM_001145946	
RUSC1-NM_001105204	LCMT1-NM_016309	ZNF200-NM_198088	FOLR2-NM_000803	
PDE2A-NM_002599	RNF214-NM_001077239	WNT5B-NM_032642	HIC1-NM_006497	
SF1-NM_004630	PRICKLE1-NM_001144883	NET1-NM_005863	PDE2A-NR_026572	
SLC25A3-NM_213611	C1orf26-NM_001105518	IRF7-NM_001572	DOCK9-NM_001130048	
DEDD-NM_001039712	TBX5-NM_080717	CDK11B-NM_033489	PTPN5-NM_032781	
ACVRL1-NM_001077401	STMN1-NM_203399	KAZ-NM_001017999	HTR7-NM_000872	
UNKL-NM_023076	ACD-NM_022914	PRR5L-NM_024841	LGALS12-NM_033101	
STMN1-NM_005563	CDK11B-NM_033488	AMICA1-NM_001098526	SIGIRR-NM_001135054	
ATP2A1-NM_173201	GFI1-NM_001127215	C1orf183-NM_019099	FES-NM_001143785	
NBL1-NM_182744	UNKL-NM_001037125	SYNE2-NM_182914	TPM1-NM_001018006	
CDK11A-NM_033529	CHD3-NM_001005271	TBX5-NM_000192	NECAP2-NM_018090	
LRRC43-NM_152759	DEDD-NM_032998	WDR20-NM_181291	MOBKL2C-NM_201403	
TPM3-NM_001043352	PML-NM_033239	SPG20-NM_015087	C15orf40-NR_027650	

SAPS3-NM_018312	XRCC3-NM_001100119	ABLIM1-NM_001003407	GLG1-NM_012201	CNTN1-NM_001843
CUL4A-NM_003589	PUS7L-NM_001098614	GAS7-NM_003644	CYB561D1-NM_001134404	PRICKLE1-NM_001144882
HNRNPR-NM_001102398	DDHD1-NM_001160148	ENTPD1-NM_001098175	RNF214-NM_207343	ACSL5-NM_203380
PUS7L-NM_031292	ABLIM1-NM_002313	TRIM34-NM_130390	PC-NM_000920	RPS6KA1-NM_001006665
KATNAL1-NM_001014380	C14orf159-NM_001102366	C1orf26-NM_017673	PC-NM_001040716	LPXN-NM_004811
AK2-NM_001625	C10orf78-NM_145247	CASP7-NM_033338	RFX5-NM_000449	LAMB3-NM_001017402
DNM1L-NM_005690	KATNAL1-NM_032116	SAPS3-NM_001164164	INPP5K-NM_130766	RAB27A-NM_183235
C12orf65-NM_152269	ASPA-NM_000049	MCL1-NM_021960	SPG20-NM_001142296	CYLD-NM_001042412
MAX-NM_145116	ITIH5-NM_030569	UBTF-NM_001076684	SLC43A3-NM_199329	
TDRKH-NM_001083965	ENTPD1-NM_001776	ARL6IP4-NM_018694	USP44-NM_001042403	

Supplementary Figure 11

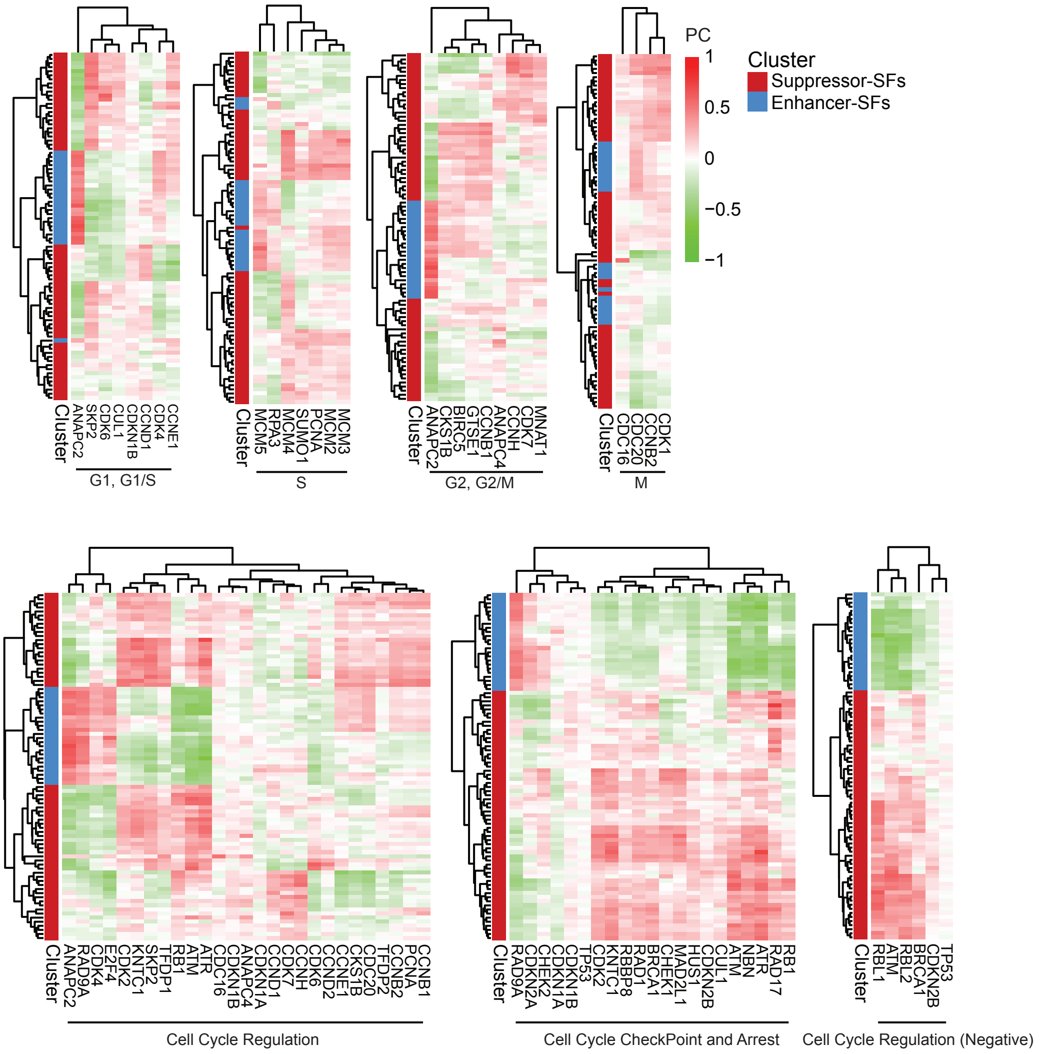

Supplementary Figure 12

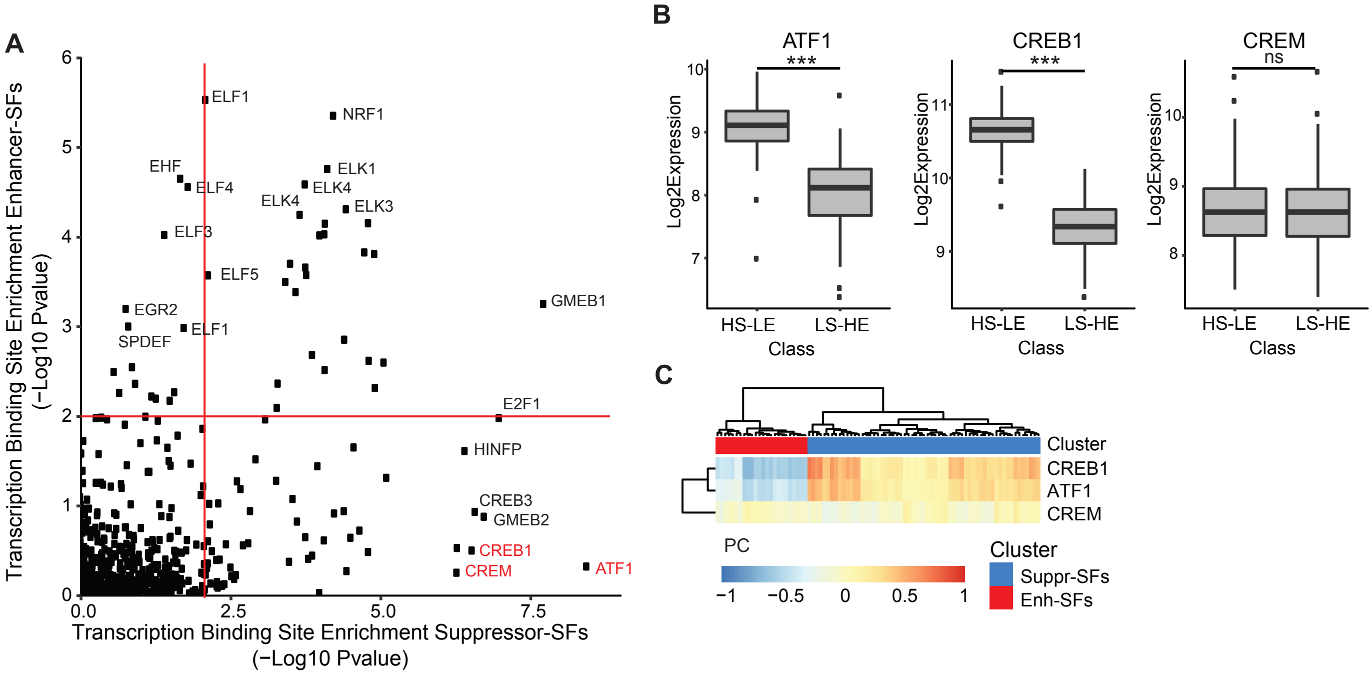

Supplementary Figure 13

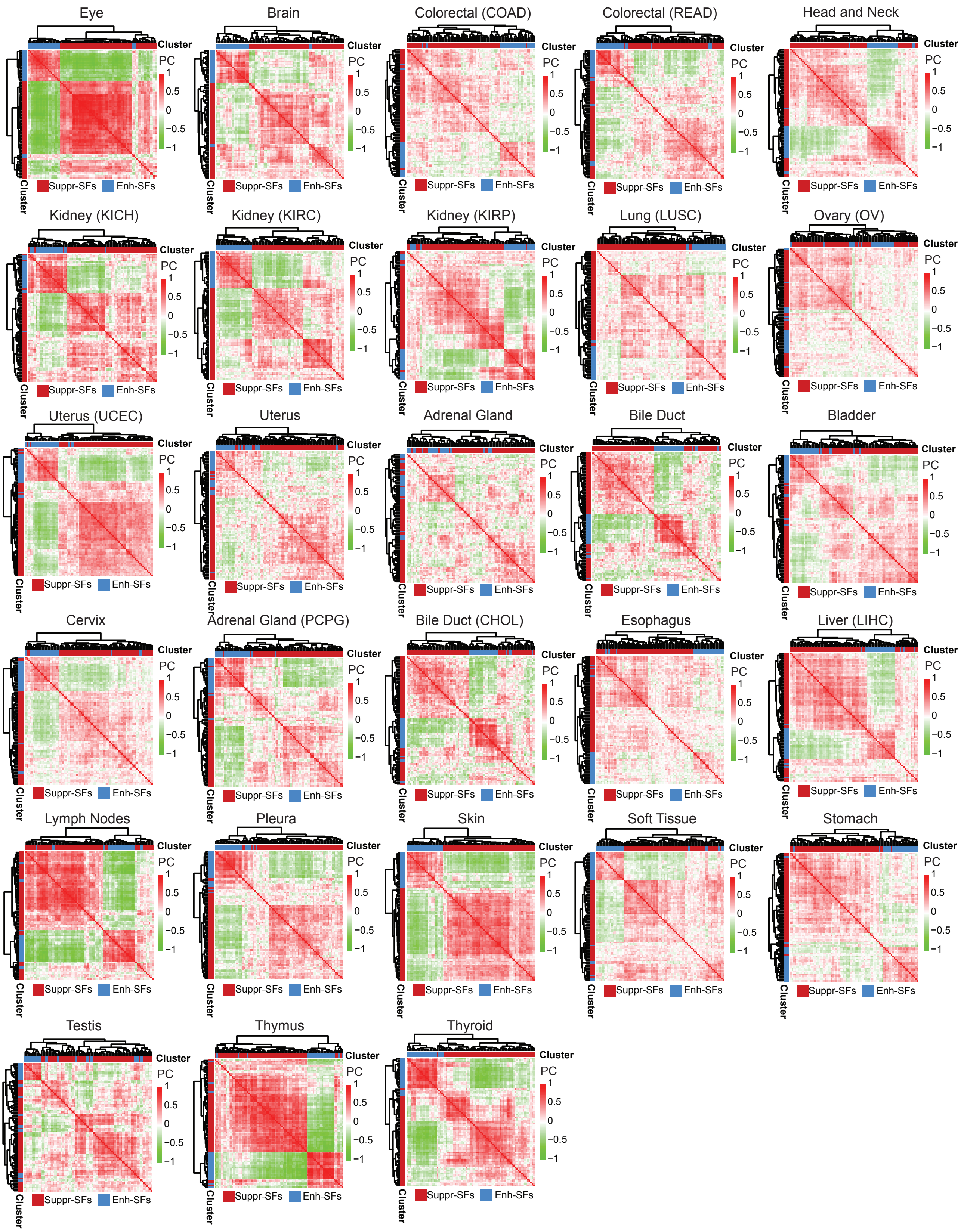

Supplementary Figure 14

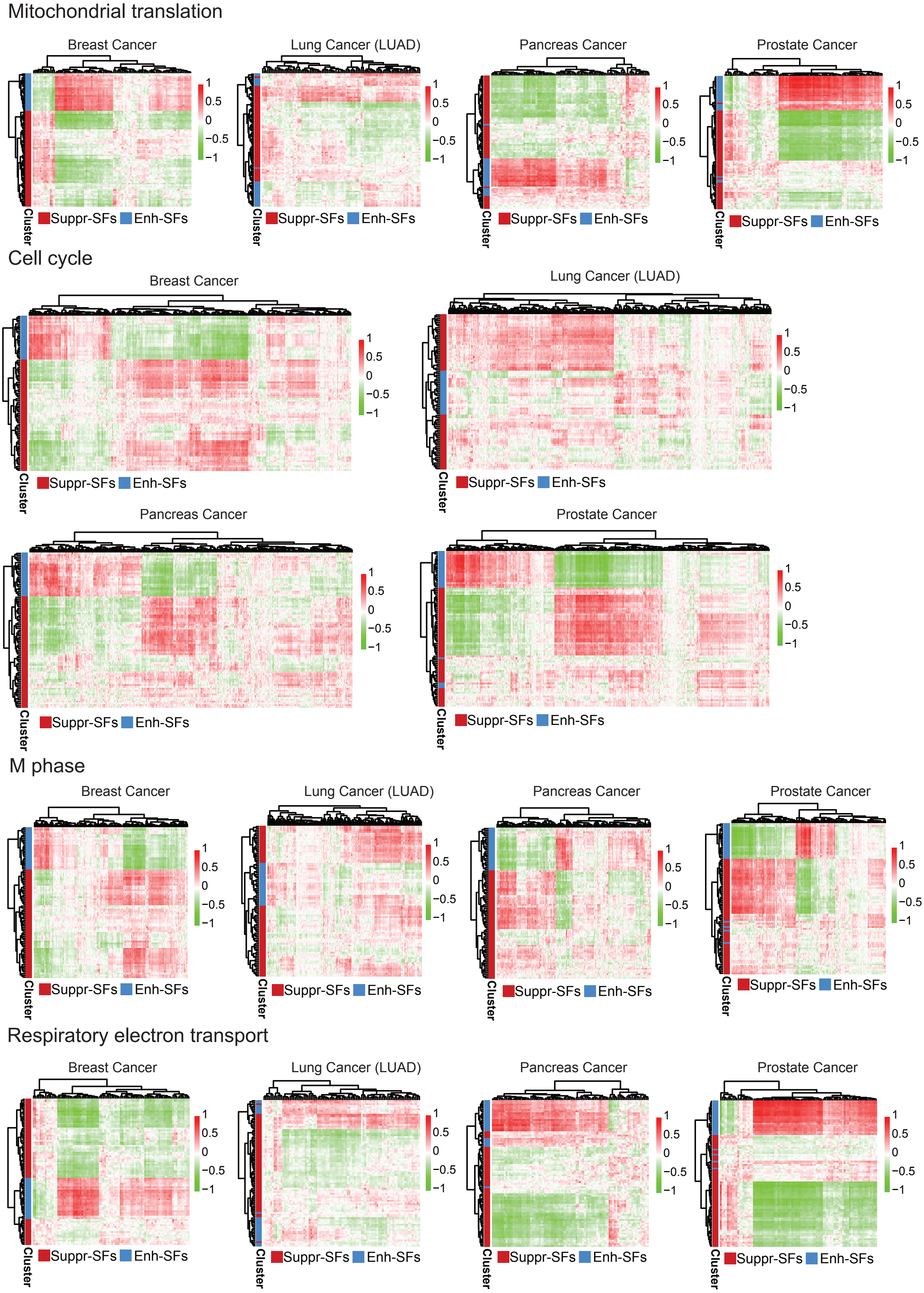

Supplementary Figure 15

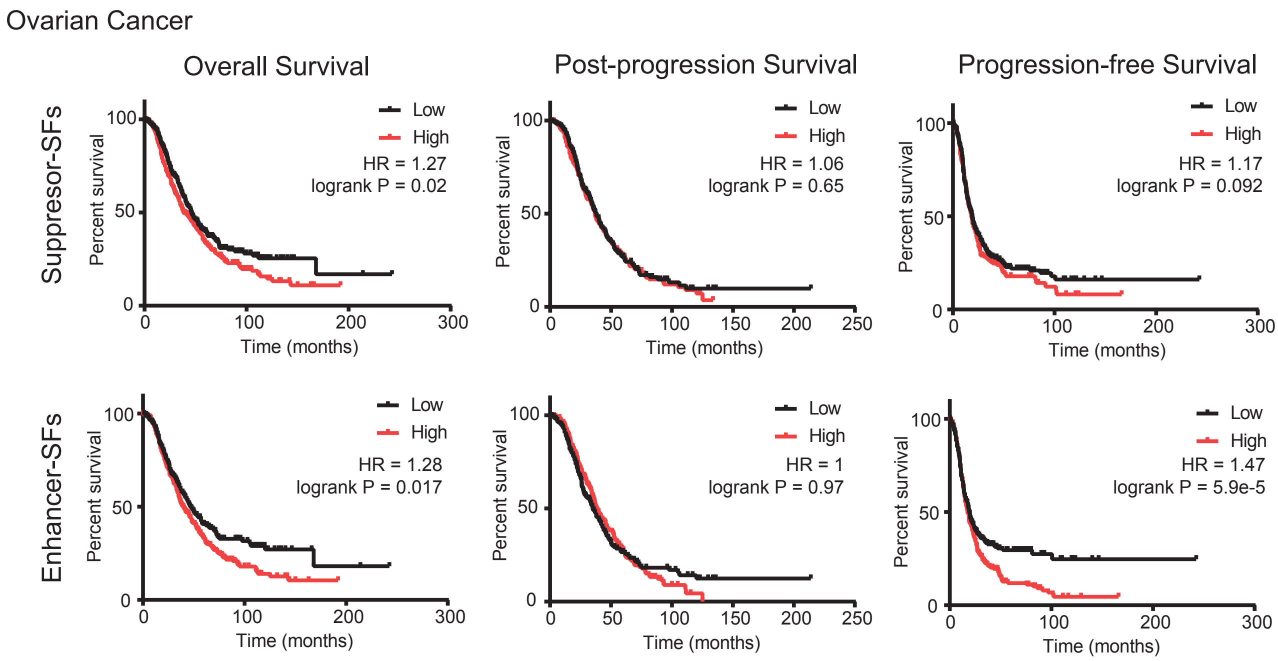

Supplementary Figure 16

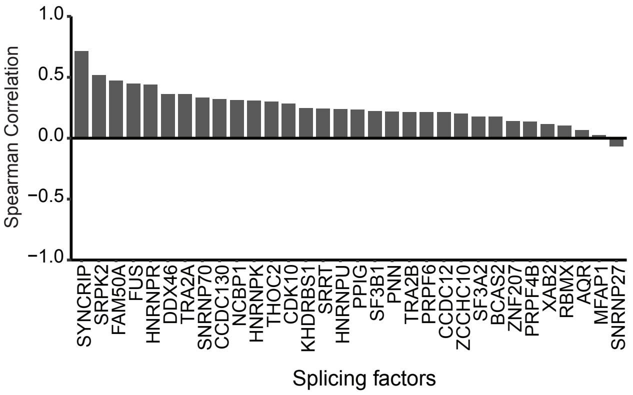

Supplementary Figure 17

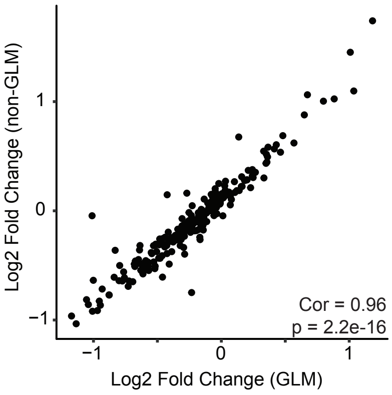

Supplement: Supplementary file 1 — Supplementary figures and legends [file 41598_2019_40759_MOESM1_ESM.pdf]
